# Hydrogen‐Induced Amorphization of Superlattice Cerium Nickel Intermetallics Enabling Efficient Alkaline Oxygen Evolution Reaction

**DOI:** 10.1002/advs.74479

**Published:** 2026-02-25

**Authors:** Ziliang Chen, Shouyu Yao, Jiaxin Xu, Xingfan Zhang, Kui Yin, Hongyuan Yang, Tianjue Hou, Ruotao Yang, Prashanth W. Menezes, Zhenhui Kang

**Affiliations:** ^1^ State Key Laboratory of Bioinspired Interfacial Materials Science Institute of Functional Nano & Soft Materials (FUNSOM) Jiangsu Key Laboratory for Carbon‐Based Functional Materials & Devices Soochow University Suzhou Jiangsu P. R. China; ^2^ Department of Material Chemistry for Catalysis Helmholtz‐Zentrum Berlin für Materialien und Energie Berlin Germany; ^3^ Department of Physics University of Basel Basel Switzerland; ^4^ Department of Chemistry: Metalorganics and Inorganic Materials Technical University of Berlin Berlin Germany

**Keywords:** amorphization, intermetallics, oxygen evolution reaction, rare earth metal, surface reconstruction

## Abstract

Transforming intermetallics with highly ordered structures into amorphous forms to unlock their superior oxygen evolution reaction (OER) remains a significant challenge. This study introduces a hydrogen‐induced amorphization approach, where hydrogen triggers severe lattice mismatches between subunits in the superlattice‐structured CeNi_3_ intermetallic, resulting in its amorphization. This process notably enhances its alkaline OER performance. Remarkably, the amorphized CeNi_3_ requires a mere 318 mV of overpotential to drive 100 mA cm^−2^, which is approximately 40 mV lower than its crystalline counterpart. Additionally, it sustains stable operation at about 620 mA cm^−2^ for 200 h in 1.0 m KOH. Comprehensive characterizations, including X‐ray absorption spectroscopy, in situ Raman spectroscopy, in situ differential electrochemical mass spectroscopy, density functional theoretical calculations, and post‐OER analyses, reveal that the amorphized CeNi_3_ undergoes a favorable surface reconstruction process, forming a Ce‐doped NiOOH active phase that follows the adsorbate evolution mechanism, thereby enhancing both the OER activity and durability of the material. The gaseous hydrogen engineering strategy outlined here offers valuable insights for designing novel rare earth‐transition metal‐based electrocatalysts, paving the way for advancements in this emerging field of electrocatalysis.

## Graphical abstract

A gaseous hydrogen engineering strategy is employed to induce the amorphization of superlattice‐structured CeNi_3_ intermetallic, facilitating alkaline OER‐driven phase reconstruction and enhancing catalytic performance.

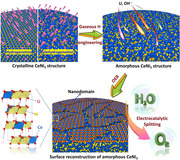

## 1 Introduction

Alkaline water electrolysis plays a critical role in hydrogen fuel production, offering significant cost advantages over acid‐based methods [1, 2]. To boost the efficiency and durability of alkaline water electrolysis, there is an urgent need to identify earth‐abundant electrocatalysts that can withstand long‐term stability while accelerating the oxygen evolution reaction (OER) kinetics inherent to the process [3]. Transition metal‐based intermetallic compounds (e.g., FeSi, Mo_6_Co_7_, NiGe) have emerged as promising precatalysts for electrochemical OER, because of their unique blend of ordered crystal structures and diverse chemical functionalities [4, 5, 6, 7, 8, 9]. In the alkaline OER environments, the surfaces of those compounds undergo a transformation, resulting in the generation of defective transition metal (oxy)hydroxides that serve as the actual catalytic phases. On the other hand, the core of these intermetallics usually retains its original structure, which is essential to produce a distinctive core‐shell architecture characterized by enhanced conductivity, ultimately optimizing charge transfer during the OER [10]. Nevertheless, most of these compounds suffer from the gradual loss of active sites under prolonged OER operation, which limits their durability and efficiency. Moreover, these reconstructed structures often lack intrinsic activity modulation, hindering further optimization. Thus, developing strategies that mitigate the dissolution of active sites and concurrently promote the formation of optimally configured transition metal (oxy)hydroxides is critical to unlock a new paradigm in the design of advanced intermetallic catalysts.

Recent pioneering studies have shown that the incorporation of rare‐earth (RE) elements into transition metal (TM) intermetallics may provide a strategic solution to address the above‐mentioned issues [11, 12, 13, 14, 15, 16]. The incorporation of RE species not only promotes the transformation of the corresponding insoluble (oxy)(hydr)oxide compounds, which helps to confine and protect the active sites [13], but also prevents the leaching of metal species that could otherwise lead to performance degradation. Moreover, the RE (hydr)oxides generate the *d‐f* electronic orbital coupling with the active TM (oxy)hydroxides [14, 15, 16], leading to intriguing charge configurations that can effectively modulate the OER activity. This approach presents a promising route to enhance both the stability and catalytic activity of OER catalysts. Despite the potential benefits of integrating RE elements, research in this domain is still in its nascent stages, and the role of RE elements in such catalytic reactions requires further investigation to fully exploit their potential in OER. Besides, the paucity of exposed active metal sites in these reported compounds remains a significant barrier to achieving optimal catalytic performance.

Amorphous engineering has gained prominence in materials science due to its ability to impart unique properties that crystalline materials cannot offer [17]. Early reports have highlighted the improved mechanical properties and corrosion resistance of amorphous metals compared to their crystalline counterparts [18, 19]. More recently, studies have unveiled the advantages of amorphization in catalysis. These findings suggest that amorphous structures can enhance catalytic performance by providing more accessible and reactive surfaces, tunable electronic properties, and higher defect sites that can serve as active centers for catalysis [20−25]. Based on these insights, it is hypothesized that amorphization RE‐TM intermetallics could substantially boost their catalytic performance. However, despite the demonstrated advantages of amorphization in various material systems, the amorphization of RE–TM intermetallic compounds for OER applications has remained largely unexplored. This gap is particularly significant given the considerable potential of these materials to enable new design strategies and performance breakthroughs in electrochemical processes.

To investigate this phenomenon, this study selects CeNi_3_ as a model system for demonstrating the proof‐of‐concept. CeNi_3_ was selected due to the following three reasons: first, Cerium is abundant, cost‐effective, and has been proven to enhance the catalytic performance of materials [4, 26−32]; second, CeNi_3_ could adsorb and desorb the hydrogen under ambient conditions, making it a promising candidate for gaseous hydrogen engineering [33], thirdly, the [CeNi_2_] subunit has a much larger volume than the [CeNi_5_] subunit, and hydrogen preferentially occupies the [Ce_2_Ni_4_] subunit, further promoting lattice misfit during hydrogenation (Figure S1) [34, 35]. Bearing the above‐mentioned points in mind, we successfully induced the amorphization of CeNi_3_ through gaseous hydrogen engineering, which significantly enhanced its alkaline OER performance compared to its crystalline counterpart. Remarkably, the amorphized CeNi_3_ demonstrates an impressive reduction in overpotential, approximately 40 mV lower than its crystalline form at a current density of 100 mA cm^−2^, and substantially outperformed pristine Ni. Furthermore, the material exhibited sustained stability, maintaining consistent operation at 620 mA cm^−2^ for an extended duration of 200 h in a 1.0 m KOH solution. Comprehensive characterization techniques, including advanced methods such as X‐ray absorption spectroscopy (XAS), in situ Raman spectroscopy, in situ differential electrochemical mass spectroscopy (DEMS), theoretical calculations, and post‐OER analyses, revealed a unique surface reconstruction process. Specifically, the surface transformed into a Ce‐doped NiOOH active nanodomain, forming a Ce‐doped NiOOH active phase that follows the adsorbate evolution mechanism, and the charge accumulated in the amorphized CeNi_3_, leading to the more favorable surface transformation. This transformation not only ensured sufficient exposure of active sites with defects but also effectively suppressed the dissolution of Ni species by the Ce‐coupling effect, thus significantly enhancing both the OER activity and durability. The gaseous hydrogen engineering strategy presented in this study offers valuable insights into the potential of RE‐TM‐based electrocatalysts. These findings serve as a solid foundation for the development of high‐performance electrocatalysts, paving the way for future advancements in energy conversion technologies.

## 2 Results and Discussion

**FIGURE 1:**
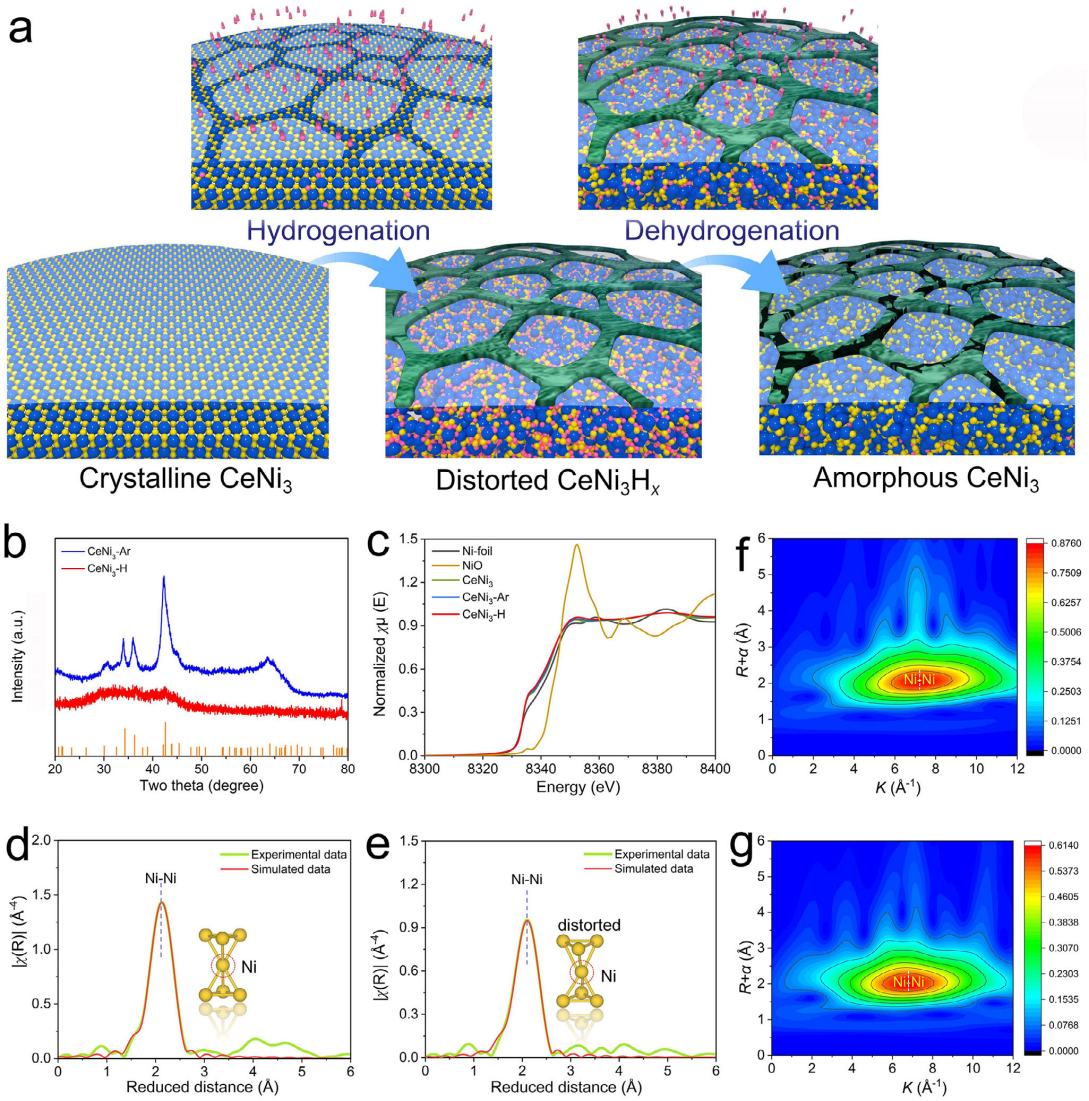
(a) The schematic diagram for the hydrogen‐induced amorphization of CeNi_3_. (b) Comparison of XRD patterns of CeNi_3_‐H and CeNi_3_‐Ar, (c) XANES spectra at the Ni *K*‐edge of Ni foil, NiO, CeNi_3_, CeNi_3_‐Ar and CeNi_3_‐H, and *k^3^*‐weighted Fourier transforms of EXAFS spectra at Ni *K*‐edge for (d) CeNi_3_‐Ar and (e) CeNi_3_‐H. Wavelet transform of the *k* ^3^ weighted Ni *K*‐edge EXAFS spectra of (f) CeNi_3_‐Ar and (g) CeNi_3_‐H.

Figure 1a schematically illustrates the process of hydrogen‐induced amorphization in CeNi_3_. When crystalline CeNi_3_ is exposed to high‐pressure gaseous hydrogen, its inherent ability to absorb hydrogen allows hydrogen atoms to be stored within the polyhedral interstitials of the CeNi_3_ lattice. This leads to an anisotropic expansion of the [Ce_2_Ni_4_] and [CeNi_5_] subunits. Due to the significant inherent mismatch at the interface between these two subunits, coupled with hydrogen atoms initially occupying the [A_2_B_4_] subunit followed by occupying the [AB_5_] subunit, sustained hydrogenation causes lattice stress. Upon subsequent hydrogen desorption, lattice contraction occurs, leading to the severely distorted CeNi_3_ structure and eventual amorphization. Notably, a small fraction of hydrogen atoms may become trapped within the interstitial sites of the CeNi_3_ lattice, though these are difficult to detect experimentally [35]. Figure S2 presents the Rietveld refinement results of the X‐ray diffraction (XRD) patterns for the pristine CeNi_3_ compound (Table S1), confirming not only the high purity of the CeNi_3_ phase but also revealing that CeNi_3_ crystallizes in a hexagonal structure with lattice parameters of *a* = 4.9681(4) Å and *c* = 16.533(1) Å. To perform the hydrogenation and dehydrogenation cycle, the as‐obtained CeNi_3_ was milled under a hydrogen atmosphere for 12 h, followed by desorption under vacuum for 30 min, and then stored under ambient conditions for further use. The cycled product is referred to as CeNi_3_‐H. For comparison, the pristine CeNi_3_ was also milled under an Ar atmosphere for the same duration, and the resulting product was designated as CeNi_3_‐Ar. Compared to pristine CeNi_3_, the XRD pattern for CeNi_3_‐Ar exhibits broadened diffraction peaks (Figure 1b), attributable primarily to the mechanical milling‐induced crystallite refinement and lattice strain. Nevertheless, most diffraction peaks remain clearly discernible, and the Rietveld refinement of the XRD pattern for CeNi_3_‐Ar suggests similar lattice parameters (Table S1) to those of pristine CeNi_3_ (Figure S3), demonstrating its mostly retained crystallinity. Figure 1b further compares the XRD patterns of CeNi_3_‐Ar and CeNi_3_‐H. Evidently, the XRD pattern for CeNi_3_‐Ar displays severely broadened diffraction peaks in a wide range, indicating its amorphous nature. X‐ray absorption spectroscopy (XAS) analysis of the Ni *K*‐edge presents the metallic feature of Ni species in both CeNi_3_‐Ar and CeNi_3_‐H, suggesting that Ni maintains its metallic valence state (Figure 1c). Corresponding EXAFS simulations reveal the presence of the Ni─Ni bonds in both crystalline CeNi_3_‐Ar and amorphous CeNi_3_‐H, suggesting their close similarities in local structure (Figure 1d,e; Figure S4). Nevertheless, the intensity of the Ni─Ni bond is significantly weaker in CeNi_3_‐H compared to CeNi_3_‐Ar and higher coordination shell signals are nearly absent in CeNi_3_‐H (Figure 1d), further demonstrating its amorphous feature. Moreover, the first coordination number for the Ni center in CeNi_3_‐H is lower than in CeNi_3_‐Ar, indicating the existence of more unsaturated coordination sites (Table S2). Besides, the Ni─Ni bond length in CeNi_3_‐H is slightly contracted compared to CeNi_3_‐Ar, implying a more severely distorted local structure (insert in Figure 1e) [35]. Note that the XAS results for CeNi_3_‐Ar are quite similar to the pristine CeNi_3_ (Figure S5 and Table S2). The local structural information extracted from EXAFS is also consistent with the corresponding Wavelet transform of the *k* ^3^‐weighted EXAFS at the Ni *K*‐edge of CeNi_3_‐H (Figure 1f) and CeNi_3_‐Ar (Figure 1g).

**FIGURE 2:**
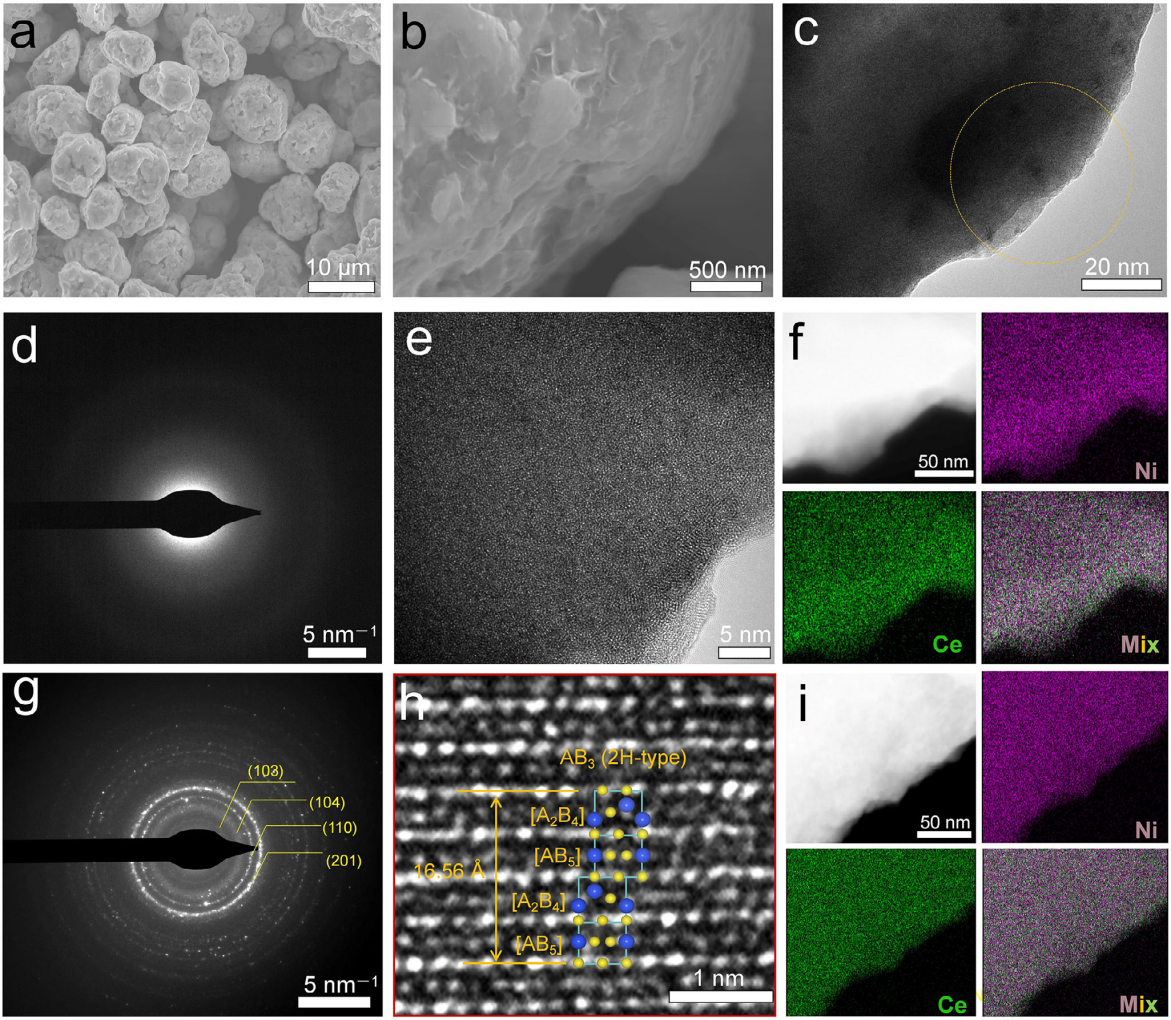
(a,b) FESEM images, (c) TEM image, (d) the corresponding SAED pattern, (e) HRTEM image, as well as (f) HAADF image of the representative region and the corresponding elemental mapping of Ni and Ce species for CeNi_3_‐H. (g) SAED pattern and (h) HRTEM image, as well as (i) HAADF image of the representative region and the corresponding elemental mapping of Ni and Ce species for CeNi_3_‐Ar.

To analyze the morphology of the product, Figure 2a,b provide field‐emission scanning electron microscopy (FESEM) images of CeNi_3_‐H, revealing a highly rough particle surface with sizes predominantly ranging from 0.5 to 40 µm. This morphology closely resembles that of CeNi_3_‐Ar, as shown in Figure S6. Moreover, compared to the pristine CeNi_3_, the particle sizes of both CeNi_3_‐H and CeNi_3_‐Ar appear significantly reduced (Figure S7), due to mechanical milling. Energy‐dispersive X‐ray spectroscopy (EDS) mapping demonstrates that the composition of Ce and Ni in all three samples closely aligns with the theoretical ratio of 1: 3 (Figures S8–S10). A low‐magnification transmission electron microscopy (TEM) image of CeNi_3_‐H, presented in Figure 2c, further corroborates the morphology observed in the FESEM images. Additionally, the corresponding selected area electron diffraction (SAED) pattern exhibits a pronounced halo at the center (Figure 2d), again indicative of an amorphous material. High‐resolution TEM (HRTEM) images uncover that the internal atoms are almost disordered, manifesting an amorphous state (Figure 2e, more TEM images can be found in Figure S11), which is in agreement with the XRD observation. Figure 2f displays the high‐angle annular dark‐field (HAADF) image along with the elemental mapping results, showcasing a consistent bright area that suggests a uniform distribution of Ce and Ni species. Additional HAADF and mapping data covering a broader region are available in Figure S12. In contrast, the SAED result for CeNi_3_‐Ar affirms its crystalline lattice structure (Figure 2g). Moreover, the HRTEM images distinctly reveal the characteristic superlattice structure of CeNi_3_‐Ar, composed of alternative stacking of [Ce_2_Ni_4_] subunit and [CeNi_5_] subunit along the *c*‐axis (Figure 2h; Figure S13). The length of *c* is estimated as 16.55 Å, being consistent with the Rietveld refinement (Figure S3 and Table S1). HAADF image and elemental mapping indicate a homogeneous distribution of Ce and Ni species (Figure 2h,i, additional HAADF and mapping data with a broader region are available in Figure S14). Furthermore, these HRTEM observations for CeNi_3_‐Ar closely resemble those of the pristine CeNi_3_ compound that we synthesized (Figure S15), suggesting that most crystalline features of CeNi_3_ are preserved in CeNi_3_‐Ar. The above collective findings successfully verify the hydrogen‐induced amorphization of the CeNi_3_ intermetallic compound.

**FIGURE 3:**
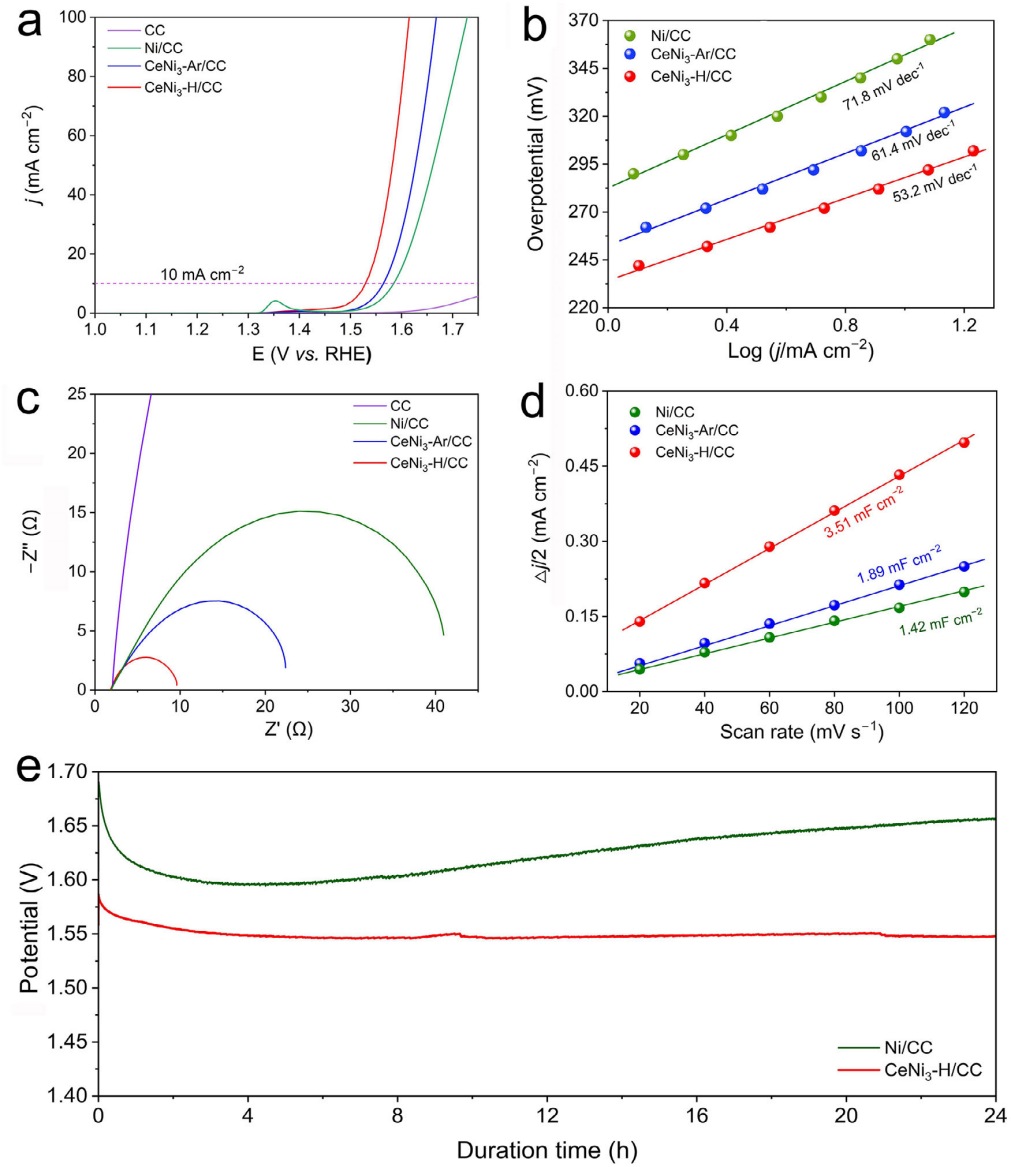
(a) Forward CV curves, (b) Tafel slopes, (c) EIS spectra, (d) C_dl_ values of activated Ni/CC, CeNi_3_‐Ar/CC, and CeNi_3_‐H/CC electrode in 1.0 m KOH. (e) Comparison of chronopotentiometry test curves of Ni/CC and CeNi_3_‐H/CC electrode in 1.0 m KOH.

Inspired by the above results and aiming to gain deeper insights into the OER mechanism in CeNi_3_‐based compounds, we conducted alkaline OER performance tests with CeNi_3_‐H powder deposited on carbon cloth (CC). These tests were performed in comparison with CeNi_3_‐Ar/CC and Ni/CC. As illustrated in Figure 3a, the forward cyclic voltammetry (CV) curves reveal that CeNi_3_‐H/CC achieves an overpotential of merely 296 mV at a current density of 10 mA cm^−2^. This value is lower than the 335 mV observed for CeNi_3_‐Ar/CC, highlighting the catalytic benefits of amorphization. Moreover, compared to nanostructured Ni/CC (354 mV at 10 mA cm^−2^), both CeNi_3_‐H/CC and CeNi_3_‐Ar/CC exhibited reduced overpotentials, indicating that the incorporation of Ce significantly boosts catalytic activity. Tafel analyses presented in Figure 3b show that activated CeNi_3_‐H/CC possesses the lowest Tafel slope (53.2 mV dec^−1^) among the three samples, indicating superior reaction kinetics. Electrochemical impedance spectroscopy (EIS) measurements confirm that the CeNi_3_‐H/CC has the lowest charge transfer resistance (*R*_ct_ = 8.617 Ω), attesting to its exceptional charge transfer kinetics (Figure 3c; Figure S16 and Table S3). The evaluation of the electrochemically active surface area (ECSA) discloses that CeNi_3_‐H/CC contains the highest ECSA value (Figure 3d; Figure S17), suggesting a greater abundance of potential active sites. Moreover, the *C*_dl‐_normalized LSV curves showed that both CeNi_3_‐H/CC and CeNi_3_‐Ar/CC displayed higher activity than that of Ni/CC, implying the higher intrinsic activity upon the incorporation of Ce species (Figure S18). Long‐term stability was assessed via chronopotentiometry (CP) testing (Figure 3e), where CeNi_3_‐H/CC delivered excellent stability in comparison to Ni/CC, exhibiting negligible degradation in activity even after 24 h at a current density of 20 mA cm^−2^. Similarly, CeNi_3_‐Ar/CC also displayed high stability during the CP testing (Figure S19). These results align well with the previous reports [36, 37], which suggest that the presence of Ce enhances the chemical and structural stability of transition metal (oxy)(hydr)oxide‐based electrocatalysts under OER conditions. Overall, the amorphization of CeNi_3_ leads to improved catalytic activity, enhanced reaction kinetics, and long durability in alkaline OER.

To further elucidate the underlying factors contributing to the remarkable activity, kinetics, and stability, we subjected the post OER (CP for 24 h at 20 mA cm^−2^) catalysts to X‐ray photoelectron spectroscopy (XPS), FESEM, and TEM analyses. The high‐resolution XPS results for Ni 2p and Ce 3d in fresh and post OER CeNi_3_‐H/CC have been provided and compared in Figure S20. Note that in addition to the signals for metallic Ni, both Ni*^n^* ^+^ and Ce^3+^ were also observed in the high‐resolution Ni 2p and Ce 3d XPS spectra of the CeNi_3_‐H compound. This phenomenon, common for transition‐metals and alloy particles, is ascribed to the presence of a passivation layer due to partial aerial oxidation [9, 38]. The results show that the chemical state of Ni has predominantly shifted to +3, and the chemical state of Ce has almost transitioned to +4, suggesting a phase reconstruction during alkaline OER. Furthermore, compared to the fresh CeNi_3_‐H, after OER CP, the peak‐to‐background intensity ratio in the high‐resolution Ce 4f XPS spectra decreased as compared to that of the high‐resolution Ni 2p XPS spectra, indicating a reduced content of Ce species near the surface. This observation aligns with prior findings, affirming that partial dissolution of Ce species occurs during the prolonged alkaline oxidation reaction [39, 40].

**FIGURE 4:**
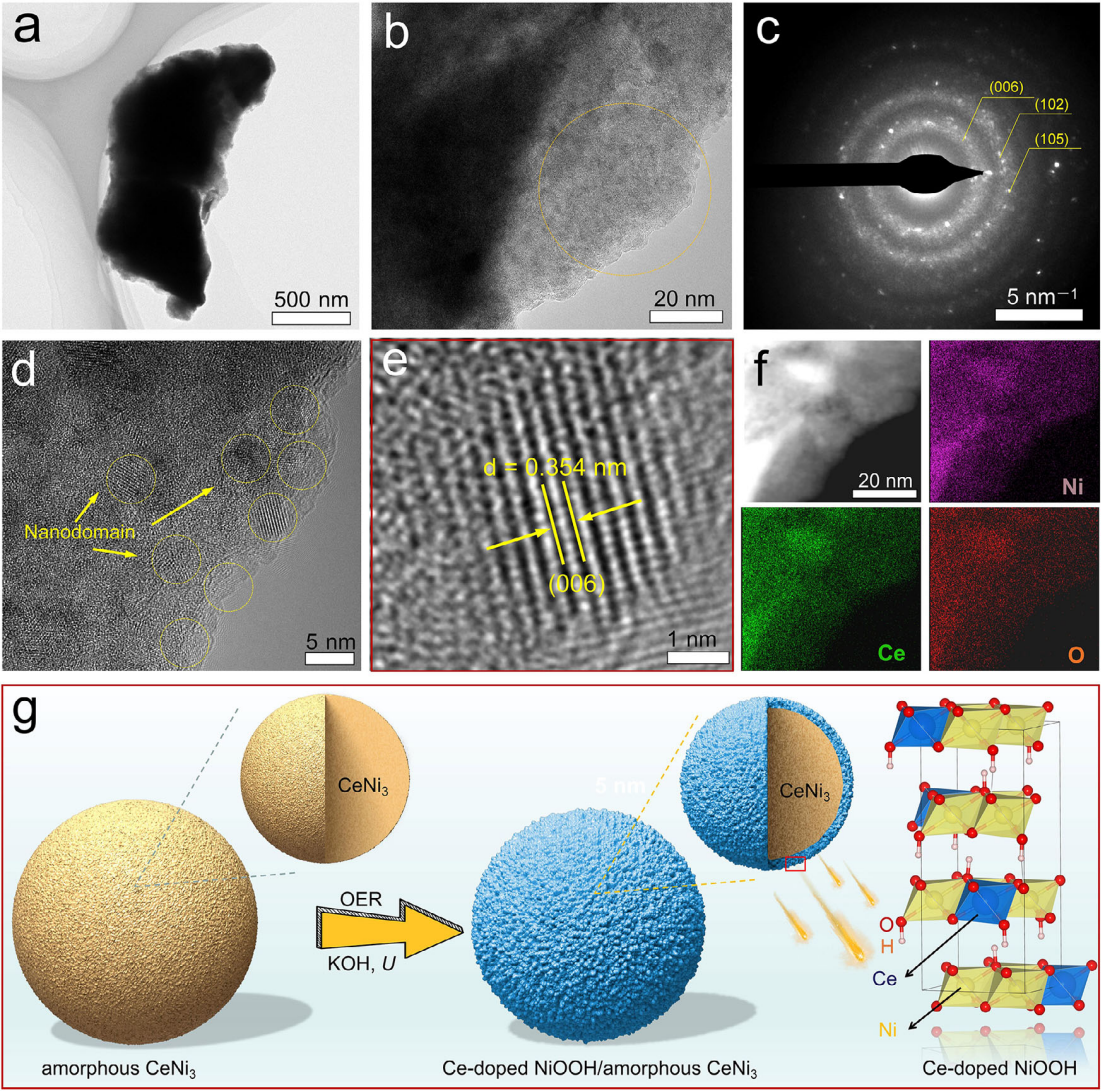
(a) TEM image, (b) magnified TEM image and (c) corresponding SAED pattern, (d, e) HRTEM images, as well as (f) HAADF image of representative region and the corresponding elemental mapping of Ni, Ce, and O species for CeNi_3_‐H after CP test. (g) Schematic illustration for the phase reconstruction of CeNi_3_‐H during the alkaline OER process.

FESEM images for post OER CeNi_3_‐H/CC uncover the emergence of a very rough structure on the surface of the particle, providing additional evidence of catalyst reconstruction (Figure S21). TEM images depict a similar morphology to that of FESEM (Figure 4a,b). However, the corresponding SAED pattern for the surface region of the particle shows the (006), (102), and (105) facets of γ‐NiOOH. Further, HRTEM analysis reveals abundant crystalline nanodomains within a shallow depth of around 10∼20 nm from the surface (Figure 4d). HRTEM identification also unequivocally establishes the crystal facet with a lattice distance of 0.354 nm as the (006) facet of γ‐NiOOH (Figure 4d), which is in accordance with the SAED results (Figure 4c). It is evident that the lattice distance of 0.354 nm is slightly larger than that (0.343 nm) of standard γ‐NiOOH (PDF#00‐006‐0075), signifying the partial substitution of Ni by the larger Ce ion. Furthermore, the HAADF image, along with its corresponding elemental mapping, elucidates a uniform distribution of Ce, Ni, and O, without obvious elemental segregation between the core and edge regions in the magnified particle (Figure 4e,f; Figure S22). Unlike the previously reported LaNi_5_ system, in which surface phase separation into LaO_x_H_y_‐confined NiOOH occurs during OER [11], the present CeNi_3_‐derived catalyst shows no detectable CeO_2_ segregation. This is further confirmed by the XRD patterns of the CeNi_3_‐H/CC electrode before and after OER chronopotentiometry (Figure S23), where, except for the peaks belonging to the carbon substrate, only the broad peaks persist without new Bragg peaks. These observations suggest the formation of a Ce‐doped NiOOH structure. Previous studies have also reported that Ce tends to form a Ce‐Ni‐OOH structure with Ni, which might be due to the smaller atomic radius of Ce compared to La, facilitating easier doping [36, 41, 42]. According to the observation and previous reports [36, 41, 42], in situ electrochemical oxidation of CeNi_3_ in 1 m KOH likely proceeds via a four‐step synergistic sequence that ultimately yields a Ce‐substituted γ‐NiOOH surface. First, anodic polarization (>1.45 V vs. RHE) selectively oxidizes Ce as Ce^3+^ while retaining Ni^2+^/^3+^ at the interface, generating Ni vacancies to accommodate the larger Ce ion. Second, Ce^3+^ is oxidized to Ce^4+^‐O and directly substitutes the [NiO_6_] octahedral site, establishing a Ce─O─Ni framework that elevates OER kinetics and thermodynamics. Third, the stronger Ce─O bond anchors the lattice, suppressing layer sliding during repeated Ni redox cycling, while Ce^4+/3+^ reversibility provides a self‐healing capability. Overall, this potential‐driven dealloying‐substitution mechanism is expected to create a robust, high‐valence surface phase that simultaneously enhances activity and structural stability. On the other hand, to check the surface composition variation, EDS spectra on the edge regions of the particle before and after OER CP were performed (Figure S24). The data revealed an atomic Ce‐to‐Ni ratio change from 1: 3 to 1: 3.9 in the edge region after OER CP. This can be due to the rapid stripping of the surface‐passivating Ce_2_O_3_‐like layer during alkaline OER [43, 44]. This transient loss inevitably biases the surface Ce/Ni ratio. This finding is further corroborated by ICP‐MS analysis (Table S4) following the post‐OER chronoamperometric test, conducted at 100 mA cm^−^^2^ for 24 h in an H‐cell (Figure S25).

Moreover, since Fe from the electrolyte may influence the OER activity of Ni‐based catalysts [45], we quantified the surface Fe content of the reconstructed phase after the chronopotentiometry test. The results show that only 1 at.% Fe was detected among all metallic species (Figure S24), confirming that Fe originating from the KOH electrolyte contributes marginally to the activity. Note that in our OER tests, we utilized KOH electrolyte with greater than 95% purity, consistent with industrial standards (85%–95%) for practical alkaline water electrolysis. Thus, the high activity and stability of the active phase can be ascribed mainly to the Ni species. Based on these insights, we propose a plausible surface reconstruction mechanism, as illustrated in Figure 4g. A similar reconstruction phenomenon was also observed for CeNi_3_‐Ar (Figure S26).

**FIGURE 5:**
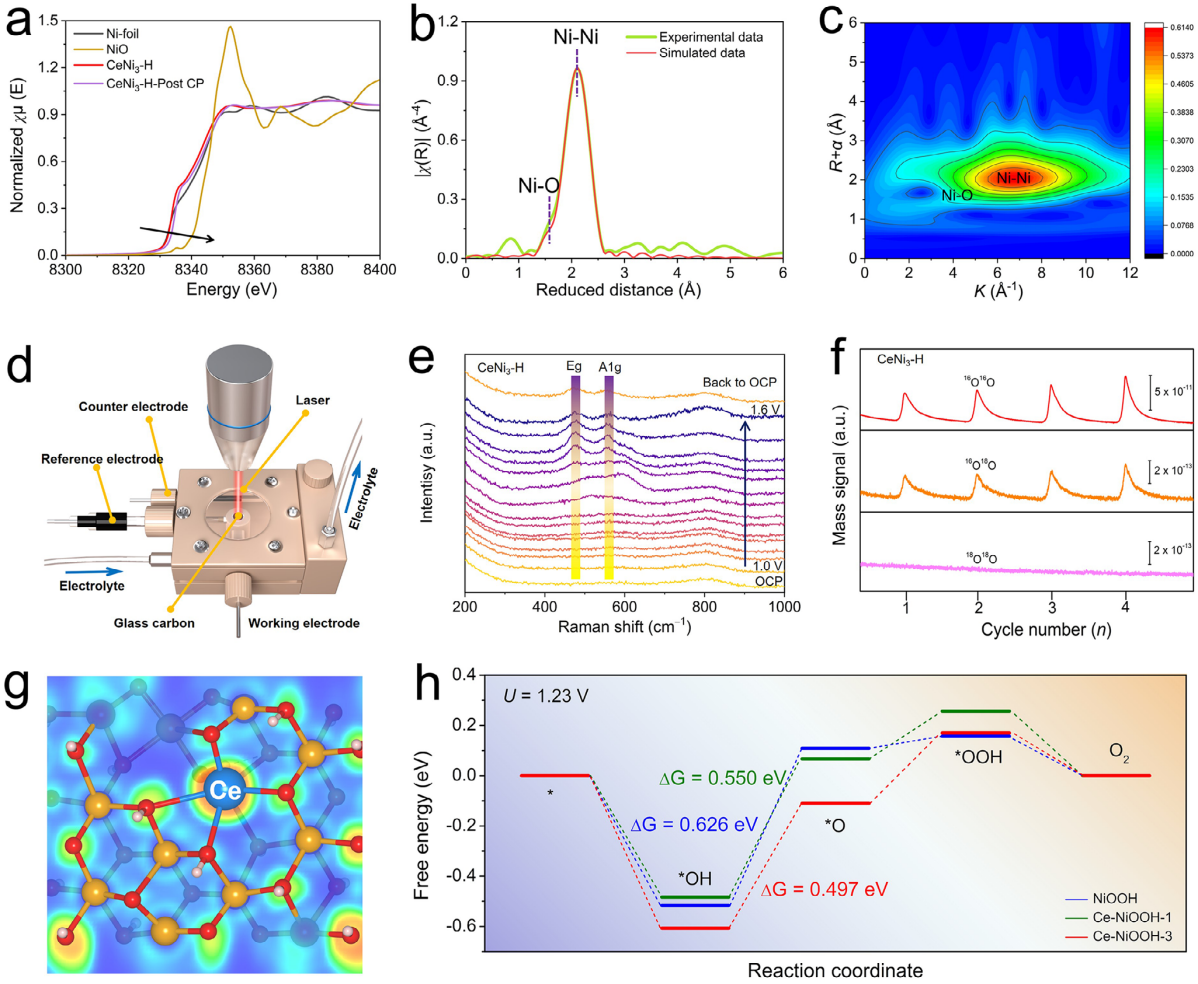
(a) XANES spectra at the Ni *K*‐edge of CeNi_3_‐H after OER CP, and (b) corresponding *k^3^*‐weighted Fourier transforms of EXAFS spectra at Ni *K*‐edge and (c) Wavelet transform of the *k* ^3^‐weighted Ni *K*‐edge EXAFS spectra. (d) Schematic illustration of the in situ Raman spectroscopy. (e) In situ Raman spectra for CeNi_3_‐H, with an increased interval voltage of 50 mV from 1.0 to 1.6 V. (f) In situ DEMS experimental data for ^18^O‐labeled CeNi_3_‐H operated in 1.0 m KOH. Four consecutive CVs are scanned from 0.25 to 0.85 V vs. Hg/HgO, while MS ion current is detected for mass channels *m*/*z*  = 32, 34, 36 (bottom to top). (g) Electron local functional map for the Ce‐contained (012) facet of Ce‐NiOOH. (h) Comparison of Free energy diagrams for 4e^−^ OER on (012) facet of NiOOH, Ce‐NiOOH‐1 and Ce‐NiOOH‐3 models at *U* = 1.23 V.

To further investigate the local structure of CeNi_3_‐H post‐OER, we conducted XAS at the Ni *K*‐edge. A slight increase in the valence state of Ni was observed (Figure 5a), implying oxidation during the catalytic process. EXAFS analyses as well as the corresponding Wavelet transform pattern of the *k* ^3^‐weighted EXAFS, revealed distinct Ni‐O and Ni─Ni coordination signatures (Figure 5b,c; Figure S27), indicative of the formation of the NiOOH phase. Inspired by the previous report on a comprehensive methodology for in‐depth structural characterization [46], we further utilized in situ Raman spectroscopy to probe the real‐time catalytic transformations to gain a deeper understanding of the catalytic mechanism (Figure 5d). The forward‐scanning Raman spectra for both CeNi_3_‐H and CeNi_3_‐Ar exhibited initial peaks around 510 and 600 cm^−1^ at 1.3 V (Figure 5e; Figure S28), which could be due to the formation of Ni(OH)_2_ [47]. Subsequently, these signals gradually attenuate, concomitant with the emergence of Ni─O vibrational bands at approximately 476 and 558 cm^−1^ (Figure 5e; Figure S28), indicative of NiOOH formation, a highly active OER phase [48]. Specifically, the band at ≈ 476 cm^−1^ corresponds to the depolarized Eg mode (in‐plane bending vibration of oxygen atoms), whereas the peak at ≈ 558 cm^−1^ arises from the polarized A1g mode (out‐of‐plane stretching vibration). Notably, CeNi_3_‐H exhibits Ni‐O oxidation at an onset potential of 1.35 V (vs. RHE, Figure 5e), which precedes that observed for CeNi_3_‐Ar (Figure S28). This suggests that the amorphous structure of CeNi_3_ facilitates alkaline OER‐driven phase reconstruction. As the reaction proceeds, the Ni─O peaks intensify, eventually evolving into a well‐defined NiOOH phase. Most importantly, even when the potential was reset to its initial state, the oxidation peak persisted, highlighting the irreversibility of the process and confirming phase reconstruction as a critical transformation. Furthermore, the relative intensity ratio of Eg to A1g peak was measured to be about 1.23 for the CeNi_3_‐H, which is lower than that (about 1.38) of CeNi_3_‐Ar, suggesting a higher density of lattice defects in the γ‐NiOOH phase of CeNi_3_‐H [48]. These results powerfully indicate that amorphization mainly enhances reconstruction kinetics, penetration depth, and defect density, rather than altering the identity of the active phase, which in turn accounts for the observed performance differences. It should be noted that a broad peak around 800 cm^−1^ can be ascribed to the signals of Nafion [49]. Subsequent transient photovoltage spectra (TPV) emphasized a pronounced broadening in the decay profile of charges on the fresh CeNi_3_‐H surface, with a delay in decay kinetics relative to CeNi_3_‐Ar (Figure S29). Such behavior suggests a stronger affinity between electrons and the catalyst surface, likely fostering enhanced interactions between metallic sites and reactants, thereby promoting the phase transformation process. Overall, compared to crystalline CeNi_3_, amorphized CeNi_3_ exhibits an increased number of defects and a higher exposure of metallic sites on its surface. This facilitates charge accumulation on the surface, thereby promoting faster reconstruction during the OER and leading to a greater number of active sites. Ultimately, this results in the formation of a heterogeneous structure where Ce‐doped NiOOH serves as the dominant active phase on the surface.

Expanding on these deeper insights, in situ differential electrochemical mass spectrometry (DEMS) experiments were conducted for identifying the reaction mechanism of CeNi_3_‐H (Figure 5f; Figure S30). Prior to DEMS testing, CeNi_3_‐H were pre‐labeled with H_2_^18^O in 1.0 m KOH (as detailed in the Experimental section) and subsequently tested in H_2_^16^O‐based 1.0 m KOH. The adsorbate evolution mechanism (AEM) and lattice oxygen mechanism (LOM) were differentiated based on mass spectrometry signals. If OER primarily follows the AEM, the mass spectrometer would predominantly detect ^16^O^16^O (*m/z* = 32) signals, with minimal ^18^O^16^O (m/z = 34) signals and no ^18^O^18^O (m/z = 36) signals. Conversely, if the LOM is dominant, all oxygen species (^16^O^16^O, ^18^O^16^O, and ^18^O^18^O) would be detected. As shown in Figure 5f, during cyclic voltammetry experiments, strong ^16^O^16^O signals (red trace) were synchronously detected with the CV cycles. Only very weak ^18^O^16^O signals (orange trace) appeared, and no ^18^O^18^O signals (pink trace) were observed. This result indicates that OER on CeNi_3_‐H mainly proceeds via the AEM. Building on this finding, density functional theoretical (DFT) calculations were performed to further explore the role of Ce in promoting OER. Specifically, three theoretical slab models were constructed, including a pristine NiOOH (012) facet and a Ce‐doped NiOOH (Ce‐NiOOH) (012) facet with less and more Ce content. Electron localization function (ELF) patterns indicate that the charge redistribution and polarization occur on the Ce‐containing surface upon the introduction of Ce into the pristine NiOOH (Figure 5g; Figure S31). The DFT calculations identify the rate determining step (RDS) of OER for NiOOH as the formation of *O intermediates (step 2, Figure S32). The largest free energy change (∆G) of the RDS in NiOOH is 1.856 eV, corresponding to a theoretical onset overpotential of 0.626 V (Figure 5h). After Ce doping, Ce‐NiOOH (Ce‐NiOOH‐1) exhibits a lower free energy change of 1.78 eV, leading to a lower theoretical onset overpotential of 0.55 V (Figure 5h). Importantly, further increasing the Ce content (Ce‐NiOOH‐3) decreases the ∆G of the RDS to 1.71 eV, highlighting the beneficial effect of Ce doping in optimizing OER kinetics. Inspired by this finding, we further probed the electronic interplay between rare‐earth elements and metallic Ni. Herein, we employed the PBE0 hybrid functional of DFT and adopted a diatomic cluster as the simplest yet representative model to investigate systematically how different alloying elements polarize and modify the electronic structure of Ni. The dopant atom was fixed at 2.5 Å from Ni, a distance commensurate with the nearest‐neighbour spacing in metallic Ni, thereby capturing the genuine interplay between outer orbitals in the alloy. Constraining the interatomic distance enables a quantitative and consistent comparison of the perturbations induced by each element. The relative polarizability produced by each dopant was evaluated from the total dipole moment of the cluster. The calculations reveal that 4f elements induce a markedly stronger polarization of Ni than 3d elements overall (Table S5). Charge‐density profiles along the internuclear axis, especially in the midpoint region, further demonstrate that 4f dopants exert a stronger disturbance on Ni's outer electrons (Figure S33), leading to enhanced orbital interaction and substantial electron redistribution, manifest as a pronounced increase in interatomic charge density. The above results provide compelling evidence that rare‐earth dopants can effectively tune the electronic structure of transition metals and thus offer an opportunity to further modulate the reconstructed RE–O–Ni active architecture.

**FIGURE 6:**
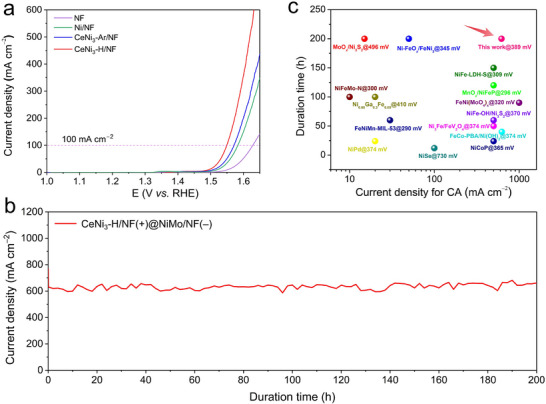
(a) Forward CV curves of activated Ni/NF, CeNi_3_‐Ar/NF, and CeNi_3_‐H/NF electrode in 1.0 m KOH, (b) Chronoamperometry test curve of CeNi_3_‐H/NF anode for overall water splitting, together with the NiMo/NF as the cathode in 1.0 m KOH. (c) Comparison of the alkaline OER performance of CeNi_3_‐H/NF with recently reported NF‐supported Ni‐based electrodes.

Motivated by these fascinating results, we applied the CeNi_3_‐H powder onto nickel foam (NF), considering its high mechanical properties and 3D electrical conductivity to further evaluate its alkaline OER performance. Notably, at a current density of 100 mA cm^−2^, the observed overpotential was a mere 318 mV, significantly lower than that of CeNi_3_‐Ar/NF and Ni/NF under analogous conditions (Figure 6a). Moreover, CeNi_3_‐H/NF demonstrated excellent durability, where it could sustain its performance for an extended period of 200 h even when subjected to a current density of about 620 mA cm^−2^ (at a cell voltage of 2.15 V, Figure 6b). The post microstructure analysis of CeNi_3_‐H (scratched from CeNi_3_‐H/NF) showed similar results to those observed in CeNi_3_‐H/FTO after the stability test (Figures S34 and S35). Such remarkable stability and catalytic efficiency rival those of the most advanced NF‐supported Ni‐based catalysts reported in recent literature (Figure 6c and Table S6), emphasizing the effectiveness of the hydrogen‐induced amorphization strategy. Notably, recent work has also shown that FeNi alloys can be reconstructed into Fe‐doped NiOOH for OER [50], mirroring the transformation observed here. Inspired by this mechanistic parallel, designing ternary rare‐earth–NiFe intermetallics may constitute a viable route toward the upper limit of OER activity and merits systematic investigation in the future. Herein, the Ce‐doped NiOOH, starting from intermetallic CeNi_3_, may offer several advantages over simple mixed oxides, LDHs or co‐precipitates: (i). Metallic core: only the outer few nanometres of CeNi_3_ are reconstructed, preserving a conductive metallic backbone that minimizes ohmic losses even at 500 mA cm^−^^2^. (ii). Scalable solid‐state route: Melting plus ball‐milling is already industrial at 100–1000 kg batches; no solvents, filtration, or freeze‐drying are needed, reducing cost and waste. (iii). Self‐limited, uniform reconstruction: electrochemical re‐insertion produces homogeneous Ce^4^^+^‐O‐Ni substitution throughout the lattice, avoiding CeO_2_/NiOOH phase separation and enhancing Ce utilization and stability. (iv). Industrial compatibility: Micron‐sized alloy powder can be plasma‐sprayed directly onto >1 m^2^ electrodes within minutes, an established process incompatible with nanosheet slurries. Encouragingly, the CeNi_3_‐H was further evaluated in an anion‐exchange‐membrane water electrolyzer (AEMWE), where it demonstrated markedly higher activity and durability than commercial Raney Ni (Figure S36). As depicted in Figure S36, it exhibits both higher activity and enhanced durability relative to commercial Raney Ni. Meanwhile, to situate this work within the evolving landscape of oxygen‐evolution catalysts, we briefly contrast it with the recent study by Park et al. [51]. They immobilize Ru through atomic Ru–Ir intermixing, a lattice‐level compositional tuning strategy. We instead trigger in‐situ reconstruction of Ce‐doped NiOOH on a Ce─Ni alloy surface, substituting spatial design for precious metals to achieve sustained oxygen evolution. The former refines atomic sites; the latter engineers interfacial architecture. These complementary approaches underscore that surface engineering alone can confer long‐term stability on precious‐metal‐free OER catalysts.

## 3 Conclusions

In summary, we have addressed the research concerns mentioned in the introduction. Specifically, we have demonstrated that hydrogen‐induced amorphization of superlattice CeNi_3_ greatly enhances charge trapping and exposure of active metal sites, accelerating surface reconstruction into the Ce‐doped NiOOH active phase. A comprehensive analysis of reaction free energies in alkaline OER further illustrated that Ce‐NiOOH possesses the most optimal reaction free energy, leading to a synergistic enhancement in catalytic activity. Upon application to highly conductive NF, the CeNi_3_‐H catalyst exhibited exceptional alkaline OER performance, achieving an overpotential of only 318 mV at a current density of 100 mA cm^−2^. This marks a substantial reduction compared to the overpotentials observed for both CeNi_3_‐Ar/NF and Ni/NF under identical conditions. Moreover, this catalyst also displayed a lower Tafel slope, indicative of its superior kinetic performance. Durability tests verified its robust performance, withstanding continuous operation for 200 h at a current density of 620 mA cm^−2^. These performance metrics highlight the efficacy of the hydrogen‐induced amorphization approach. This work not only advances our understanding of OER catalysis but also paves the way for the design of more efficient and durable electrocatalysts, holding significant promise for next‐generation energy conversion technologies.

## Conflicts of Interest

The authors declare no conflicts of interest.

## Supporting information




**Supporting File**: advs74479‐sup‐0001‐SuppMat.docx.

## Data Availability

The data that support the findings of this study are available from the corresponding author upon reasonable request.
