# Supplementary material for: Hydrogen‐Induced Amorphization of Superlattice Cerium Nickel Intermetallics Enabling Efficient Alkaline Oxygen Evolution Reaction
Source: Adv Sci (Weinh). 2026 Feb 25;13(24):e74479. doi: 10.1002/advs.74479 (PMC13116014; doi:10.1002/advs.74479)
Supplement: Supplementary file 1 — Supporting File: advs74479‐sup‐0001‐SuppMat.docx. [file ADVS-13-e74479-s001.docx]

Supporting Information

***Hydrogen-Induced Amorphization of Superlattice Cerium Nickel Intermetallics Enabling Efficient Alkaline Oxygen Evolution Reaction***

*Ziliang Chen,* Shouyu Yao, Jiaxin Xu, Xingfan Zhang, Kui Yin*, Hongyuan Yang, Tianjue Hou, Ruotao Yang, Prashanth W. Menezes*, Zhenhui Kang**

Dr. Z. Chen, S. Yao, J. Xu, Dr. K. Yin, R. T. Yang, T. Hou

State Key Laboratory of Bioinspired Interfacial Materials Science, Institute of Functional Nano & Soft Materials (FUNSOM), Jiangsu Key Laboratory for Carbon-Based Functional Materials & Devices, Soochow University, 199 Ren'ai Road, Suzhou, 215123, Jiangsu, PR China.

E-mail: [zlchen@suda.edu.cn,](mailto:zlchen@suda.edu.cn,) zhkang@suda.edu.cn

Dr. Z. Chen, Dr. H. Yang, Dr. P. W. Menezes

Department of Material Chemistry for Catalysis, Helmholtz-Zentrum Berlin für Materialien und Energie, Albert-Einstein-Str. 15, 12489 Berlin, Germany

E-mail: prashanth.menezes@helmholtz-berlin.de

Dr. X. Zhang

Department of Physics, University of Basel, Klingelbergstrasse 82, CH-4056 Basel, Switzerland

Dr. P. W. Menezes

Department of Chemistry: Metalorganics and Inorganic Materials, Technical University of Berlin, Straße des 17 Juni 135. Sekr. C2, 10623 Berlin, Germany

**Experimental section**

**Chemicals**

All chemical reagents (analytical grade) were directly used as received without further purification. Commercially available Ce (purity: 99.9%) and Ni (purity: 99.99%) metal pellets are purchased from Shanghai Titan Scientific Co., Ltd. Pure Ni powder is obtained from Hangzhou Xinchuan New Material Co., Ltd. Commercially available potassium hydroxide pellets (> 90%) were purchased from Aladdin. The carbon cloth (CC) and nickel foam (NF) were purchased from Taiwan CeTech Co., Ltd., and Kunshan Guangjiayuan New Material Co., Ltd., respectively, and used as the electrode substrate after cleaning or activation treatment. Deionized water was utilized for the whole experiment.

**Synthesis of the precatalysts**

The CeNi_3_ alloy was prepared by induction melting of appropriate amounts of Ce and Ni metals under an argon atmosphere (about 0.06 MPa). About 2 wt.% of Ce was excessively added to compensate for the loss of Ce during melting. In order to ensure the composition homogeneity, the samples were remelted three times. The samples obtained were sealed in a quartz tube filled with an Ar atmosphere and then annealed at 1023 K for one week. Finally, the as-annealed ingot was polished to remove the oxide layer and crushed into powders of less than 45 μm for further use. For hydrogenation, 3 g of CeNi_3_ alloy powder was mixed with the stainless steel balls in a weight ratio of 1 to 15 in a stainless steel vial. Then, the ball milling was performed using a QM-3SP2 planetary mill at a rotation speed of 400 rpm under a 4 MPa hydrogen atmosphere for 12 hours. During the milling process of each sample, the milling was repeated twice at 15-min intervals, with each session lasting 30 min. Finally, the hydrogenated samples were degassed for 30 min under vacuum conditions and then kept under ambient conditions for use. For comparison, 3 g of CeNi_3_ alloy powder was also milled under an Ar atmosphere for 12 h. Note that the mill we used is not equipped with in-situ temperature/pressure logging, also a limitation common to most mechanical-ball-milling set-ups, and all experiments were performed at room temperature.

**Characterization of the catalysts**

**X-ray Diffraction.** To determine the phase composition and crystal structure, powder X-ray diffraction (PXRD) was performed using a Bruker AXS D8 Advance automatic diffractometer, which was equipped with a curved germanium (111) primary monochromator and a position-sensitive detector. The PXRD pattern was subsequently refined using the RIETAN-2000 program, based on the Rietveld method.[S1] The three-dimensional visualization of the crystal structure of the synthesized compound was achieved with the VESTA program, version 3.0. [S2]

**X-ray photoelectron spectroscopy.** To obtain the chemical state information, an X-ray photoelectron spectrometer (XPS) was used to characterize the catalysts before and after OER. The XPS measurements were carried out on an ESCALAB 250Xi spectrometer (Thermo Scientific, USA) equipped with a pass energy of 30 eV with a power of 100 W (10 kV and 10 mA) and a monochromatized AlKα X-ray (hν=1486.65 eV) source. All samples were analyzed under a pressure of less than 1.0×10^-9^ Pa. Spectra were acquired through the Avantage software (Version 5.979) with a step of 0.05 еV. All the as-obtained data were fitted by XPSPEAK software. The high-resolution XPS spectra for C 1s, O 1s, Ni 2p, and Ce 3d levels were collected with a pass energy of 30 eV. The binding energies were then calibrated against the C 1s peak energy position (285.0 eV).

**Elementary Analysis.** The chemical composition of CeNi_3_ and hydrogen-processed CeNi_3_ catalysts before and after the OER durability test was accurately determined using inductively coupled plasma atomic emission spectroscopy (ICP-AES) analyses. Specifically, the catalysts were digested in aqua regia, which is a mixture of HNO_3_ and HCl in a volume ratio of 1:3. In each experiment, 2.5 mL of the digestion solution was diluted with deionized water to a final volume of 15 mL. Calibration curves were obtained for all elements, including Sm and Co, using standard solutions with concentrations ranging from 1 mg L^–1^ to 100 mg ^L–1^ (1000 mg L^–1^ Single Element ICP-Standard Solution ROTI®STAR). The average value was then calculated from three independent experiments.

**Electron Microscopy.**

We used field emission scanning electron microscopy (FESEM) to characterize the morphology of the catalyst before and after OER. A deeper understanding of the catalyst microstructure was obtained by transmission electron microscopy (TEM) using a FEI Tecnai G2 20 S-TWIN transmission electron microscope (FEI, Eindhoven, Netherlands) equipped with a LaB_6_ source at an accelerating voltage of 200 kV. EDX analysis was performed using an EDAX R-TEM SUTW detector (Si(Li) detector). A GATAN MS794 P CCD-camera was utilized to capture the images. Raman spectra.

**Raman Spectroscopy:** The in situ Raman spectra were excited by 532 nm emission emitted by an argon-ion laser and recorded using a confocal Raman spectrometer equipped with a liquid nitrogen-cooled charge-coupled device (CCD) camera as a detector (Horiba LabRam HR Evolution). The measurements were collected on a probed catalyst membrane, which was fabricated by depositing modified CeNi_3_ powder on the surface of a carbon glass electrode. The Raman signal was recorded from the initial state of the prepared CeNi_3_-H and CeNi_3_-Ar film and its in situ OER reaction state (1.0 to 1.6 V vs. RHE) in 1.0 M KOH with a voltage interval of 0.05 V.

**Transient photo-induced voltage (TPV) principle**

Transient Photovoltaic (TPV) measurements were carried out using a homemade measurement system. In this particular setup, a platinum mesh - covered powder sample (1 × 1 cm⁻^2^) served as the working electrode, and a platinum wire was the counter electrode. All the measurements were performed at room temperature. The powder samples were irradiated by a radiation pulse from a third - harmonic Ndlaser (Polaris II, produced by New Wave Research, Inc.), with the pulse having a wavelength of 355 nm and a width of 5 ns. The photocurrent that was generated went through a process of being recognized and amplified, and finally, an oscilloscope recorded it as the ratio of the photovoltage to the internal resistance of the test system.

**X-ray absorption spectroscopy.** The XAS spectra, including X-ray absorption near-edge structure (XANES) and extended X-ray absorption fine structure (EXAFS) ones, of the probed samples at Ni *K*-edge were collected at the Beamline of TLS07A1 in the National Synchrotron Radiation Research Center (NSRRC), Taiwan. Data reduction, data analysis, and EXAFS fitting were performed and analyzed with the Athena and Artemis programs of the Demeter data analysis packages that utilize the FEFF6 program to fit the EXAFS data. The energy calibration of the sample was conducted through a standard Ni foil, which, as a reference, was simultaneously measured. A linear function was subtracted from the pre-edge region, then the edge jump was normalized using Athena software. The χ(k) data were isolated by subtracting a smooth, three-stage polynomial approximating the absorption background of an isolated atom. The *K*^2^-weighted χ(k) data were Fourier transformed after applying a Kaiser-Bessel window function (Δk =1.0). For EXAFS modeling, the global amplitude EXAFS (CN, R, σ^2^ and ΔE0) was obtained by nonlinear fitting, with least-squares refinement, of the EXAFS equation to the Fourier-transformed data in R-space, using Atemis software, EXAFS of the Fe foil is fitted and the obtained amplitude reduction factor S_0_^2^ value (0.701 was set in the EXAFS analysis to determine the coordination numbers (CNs) in the Ni-O/Ni scattering path in sample.

**Electrochemical measurements**

Electrochemical measurements were conducted in a standard three-electrode electrochemical cell in a 1.0 M KOH aqueous electrolyte using an EC Lab v10.20 software package-directed potentiostat (SP-150e, BioLogic Science Instruments). The substrates (CC and NF) deposited with catalyst films served as the working electrodes, while a Hg/HgO (CH Instruments, Inc.) and a Pt foil (10 mm length × 10 mm width × 0.3 mm thick) were used as the reference and counter electrode, respectively. The electrode area was 0.5 x 1.0 cm^2^ and the mass loading for each working electrode was 4 mg cm^−2^. For the preparation of the electrode, a catalyst ink containing 12 μL of ethanol, 2 μL of Nafion, and 2 mg of catalyst powder was carefully cast onto the substrate surface and allowed to dry naturally. Cyclic voltammetry (CV) and linear sweep voltammetry (LSV) were carried out at a low scan rate of 5 mV s^−1^ and compensated with an applied iR compensation of 90%. We calibrated the potentials measured in this work to the reversible hydrogen electrode (RHE) based on the following equation in 1.0 M aqueous KOH: E(RHE) = E(Hg/HgO) + (0.059 × pH) V + 0.098 V. The Tafel slope was calculated using the steady state method, which was conducted using the potentiostatic measurements with a potential stepwise increase of 15 mV. The current density at each potential was recorded for 3 min, and its appropriate value was adopted for the determination of Tafel plots. The Tafel slope was determined based on the Tafel equation: *η* = *a* + *b*log *j*, in which *η*, *b*, and *j* mean the overpotential (V), Tafel slope (mV dec^−1^), and current density (mA cm^−2^), respectively. The electrochemically active surface area (ECSA) of all the measured samples was reflected by estimating their double-layer capacitance (C_dl_) and was tested by conducting the CV cycles at the potential range where no faradaic process was involved. The electrochemical impedance spectroscopy (EIS) was recorded at 1.53 V vs. RHE for the samples deposited on CC. During the test, the amplitude of the sinusoidal wave was defined in the frequency range from 100 kHz to 1 mHz. The diameter value of the semicircle in the Nyquist plots was extracted as the charge transfer resistance (Rct). The half difference of current density at the middle of the potential of the CV cycle was plotted as a function of the corresponding scan rate, resulting in the associated slope. The chronoamperometric measurements (CA) were performed in 1.0 M aqueous KOH to evaluate the long-term stability at the desired constant potentials against RHE.

**Theoretical calculations**

In the present work, the first-principles calculations were applied using the Vienna ab initio software package (VASP) with the projector augmented wave method[S3-S5]. Meanwhile, the generalized gradient approximation (GGA) within the Perdew-Burke-Ernzerhof (PBE) was adopted to compute the electron exchange-correlation energy.[S6] The structure was relaxed via a cutoff energy of 450 eV. The atomic position, cell volume, as well as cell shape, were fully optimized until the forces were lower than 0.01 eV Å^–1^. In the present calculation, a (3×1) unit cell was used to model the surface of γ-NiOOH (012). Based on this surface model, another two models with the atomic ratio of Ce to Ni as 1 : 11 and 1 : 3 were constructed. The 20 Å vacuum layer was normally added to the surface to eliminate the artificial interactions between periodic images, and the (3 × 3 × 1) grid was employed for *K*−space sampling during all the calculations.

The OER mechanism is considered to proceed along the following four processes in alkaline media [S7]:

* + OH → OH* + e^–^  (1)

OH* + OH → O*+ H_2_О +e^–^ (2)

O* + OH → OOH* + e^–^ (3)

OOH* + OH → Ο_2_ (g) + * + H_2_O + e^–^ (4)

where the symbol “*” refers to the active site on the catalyst.

ΔE_o*_ = E(O* - E(*) - [E(H₂O)-E(H_2_)] (5)

ΔE_oн*_ = E(OOH* - (*) - [E(H_2_O)- E(H_2_)/2] (6)

ΔE_OOH*_ = E(OH* - E(*) - [2E(H_2_O)- 3/2E(H_2_)] (7)

where E(O*), E(OH*), and E(OOH*) denote the total energy of O, OH, and OOH adsorbed on the surfaces, respectively; E(*), E(H_2_O), and E(H_2_) corresponds to the energy of the clean surface, water, and hydrogen in the gas phase. As a result, the Gibbs free energy change (ΔG) related to processes (1)-(4) was calculated by:

ΔG = ΔE + ΔZPE - TΔS (8)

In which ΔE, ΔZPE, and TΔS are the adsorption energy, change of zero point energy, and entropy contribution. The ZPE correction was obtained from vibrational frequency calculations. In addition, the ΔG*_U_* = -e*U* is the term to consider the external bias (*U* =0 and 1.23V). The free energy of O_2_ was obtained by referring to the 2H → O_2_ + 2H_2_ reaction with a free energy change of 4.92 eV.

**Computational details for the electronic interaction between the rare earth and Ni**

To quantify the electronic interaction and orbital polarisation induced by different dopant elements on Ni, a diatomic model was employed. A single Ni atom was paired with a 3d- or 4f-element atom and separated at a fixed distance of 2.5 Å, corresponding to the typical nearest-neighbour distance in metallic bulk Ni. This fixed-distance setup focuses on the intrinsic electronic response of Ni to different dopant atoms, allowing a direct comparison of effects from 3d and 4f elements.

All calculations were performed using density functional theory (DFT) as implemented in the NWChem package.[S9] The PBE0 hybrid exchange–correlation functional was used to provide an accurate description of electronic states, particularly necessary for 3d and 4f electrons.[S10] The def2-TZVP [S11, S12] basis sets, together with Stuttgart energy-adjusted pseudopotentialsfor heavy elements, were employed consistently for both Ni and all dopant atoms.[S13, S14] Calculations were performed as isolated single-point electronic structure evaluations in vacuum without any periodic boundary conditions. For each Ni–M pair, all accessible spin multiplicities were examined, and the electronic configuration of lowest energy was selected for subsequent analysis.

Dopant-induced polarisation at the Ni site was characterised by two approaches. First, the charge density along the Ni–M bond axis was extracted using the DPLOT module of NWChem, enabling a direct comparison of dopant-induced charge redistribution. Second, the relative polarisation strength was quantified from the atomic multipole expansion. Dipole components projected onto the Ni–M bond axis were extracted from the multipole analysis of the charge density, which were then used to determine the effective polarisation generated by the dopant.

**Anion Exchange Membrane Water Electrolyzer (AEMWE) Testing**

A lab-scale anion exchange membrane water electrolyzer (AEMWE) was constructed, consisting of an anode and cathode (each with a geometric area of 4.0 cm^2^), an anion exchange membrane (MTCP-50), sealing gaskets, flow fields, current collectors, and end plates. Prior to assembly, the membrane was preconditioned by soaking in 1 M KOH for a minimum of 24 hours. The electrodes were fabricated using a catalyst-coated substrate (CCS) approach. For the cathode, an ink was prepared by dispersing 200 mg of Pt/C catalyst and 350 mg of 5 wt.% ionomer in 16 g of solvent (DI water to isopropanol mass ratio of 3:8), followed by 1 hour of ultrasonication at ambient temperature. The anode ink was similarly prepared by mixing 200 mg of CeNi_3_-H with 350 mg of 5 wt.% ionomer in 16 g solvent (DI water: isopropyl alcohol = 1: 10 at mass ratio), and sonicating for 1 hour. Both inks were subsequently spray-coated onto nickel felt substrates using an ultrasonic sprayer, achieving catalyst loadings of 1 mg cm⁻^2^ for the cathode and 1 mg cm⁻^2^ for the anode. The membrane electrode assembly (MEA) was formed by sandwiching the cathode, membrane, and anode between two titanium bipolar plates, which were tightened to a torque of 8 N·m. Fluoroelastomer gaskets (3 mm thick) were used on both sides to prevent excessive compression of the electrodes. Eight bolts were tightened in a diagonal sequence to ensure uniform sealing. For comparative evaluation, a commercial Raney Ni-coated Ni mesh was also employed as the anode. The electrolyte, 1 M KOH, was circulated at 35 mL min⁻¹, and the system was operated at approximately 60 °C. Polarization curves were recorded using a Fueiceel® DC Power Supply (Model F3020).

**Figures**

**
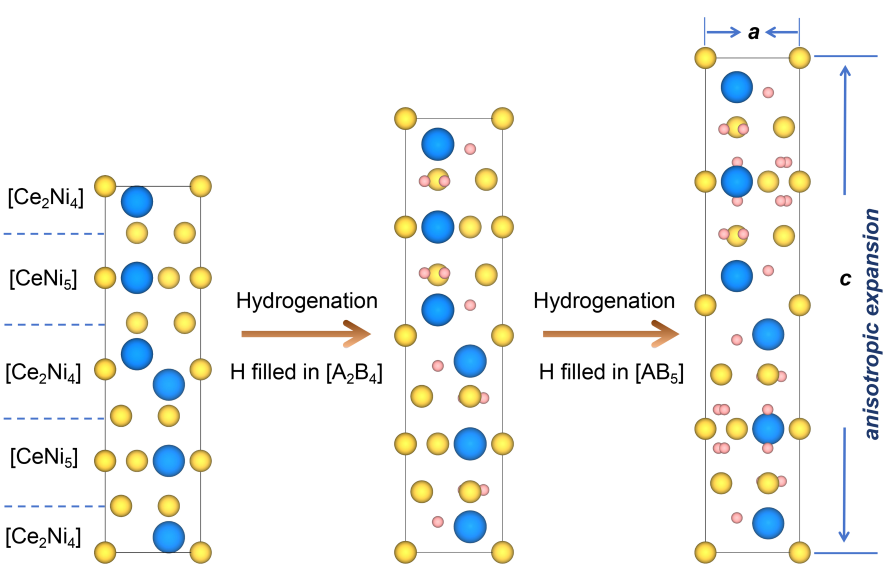
**

**Figure S1**. The schematic illustration for the lattice expansion of CeNi_3_ during hydrogenation.


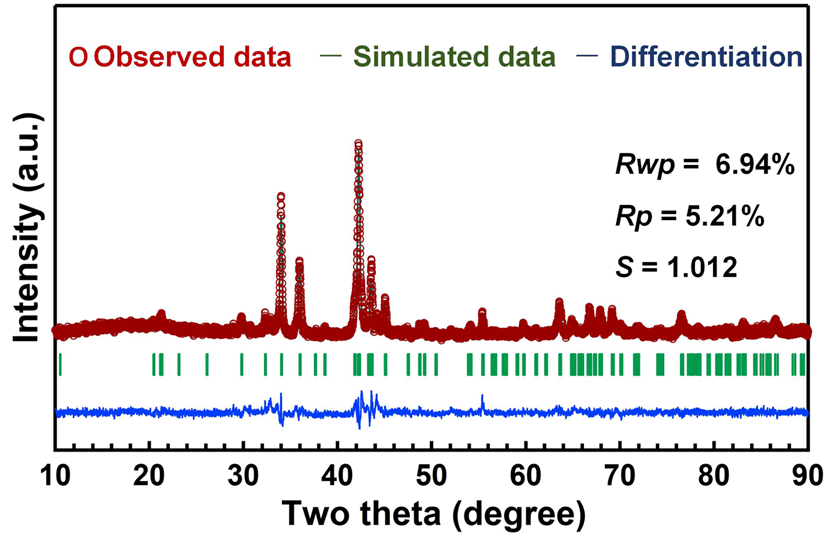


**Figure S2.** The Rietveld refinement for the XRD pattern of the pristine CeNi_3_ compound.


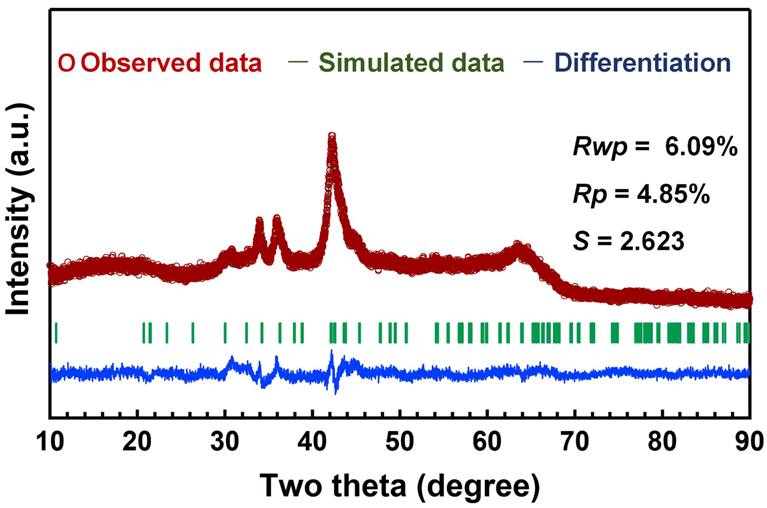


**Figure S3.** The Rietveld refinement for the XRD pattern of the CeNi_3_-Ar compound.


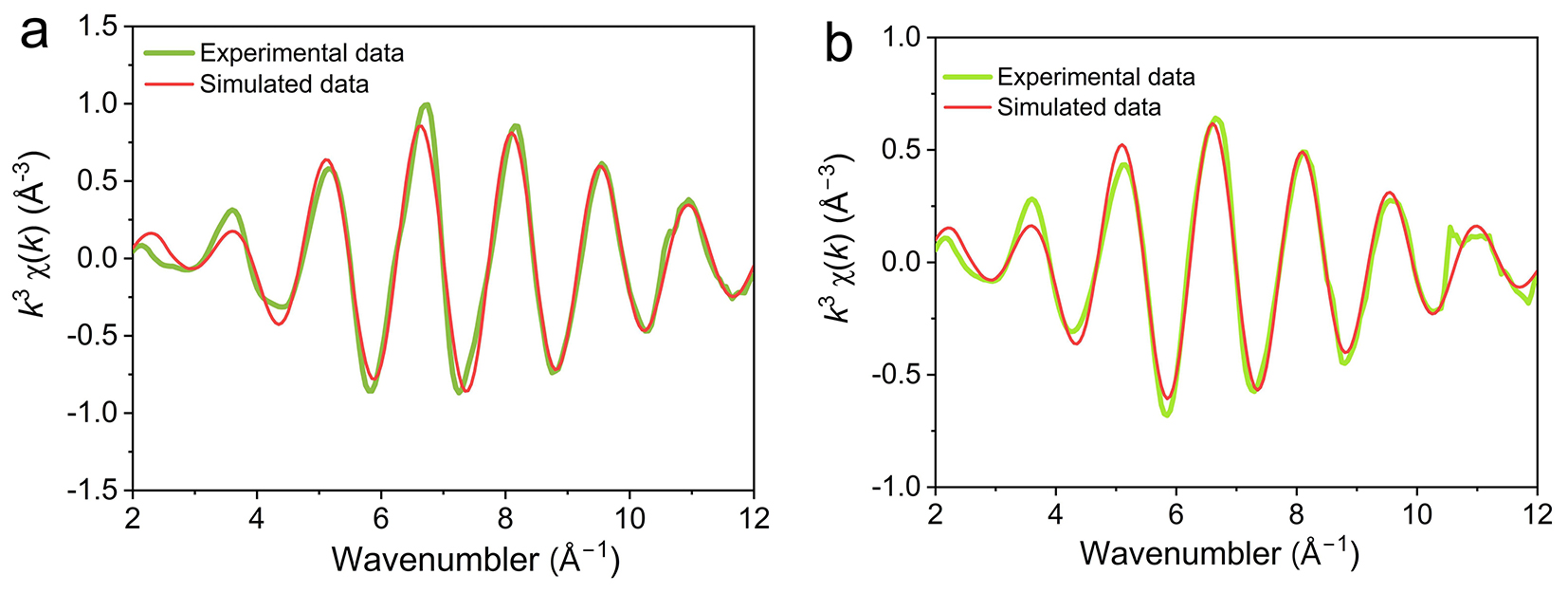


**Figure S4**. (a) *k*^3^-weighted EXAFS spectra of Ni *K*-edge for the (a) CeNi_3_-Ar and (b) CeNi_3_-H samples.

**
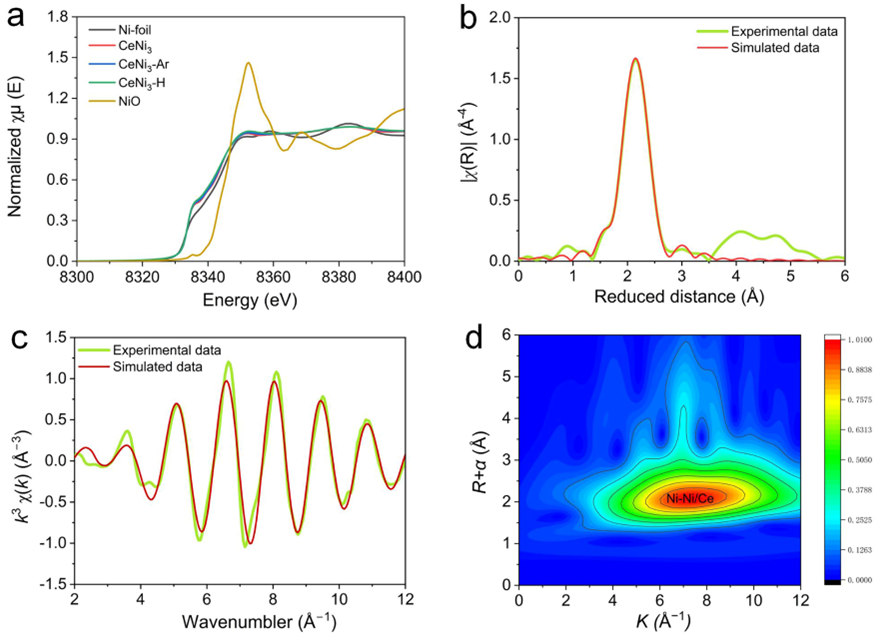
**

**Figure S5**. (a) XANES spectra at the Ni *K*-edge of Ni foil, NiO, pristine CeNi_3_, CeNi_3_-Ar, and CeNi_3_-H, and (b) *k^3^*-weighted Fourier transforms of EXAFS spectra and (c) *k^3^*-weighted Fourier EXAFS spectra of Ni K-edge for pristine CeNi_3_. (d) Wavelet transform of the *k*^3^-weighted EXAFS spectra of Ni K-edge for pristine CeNi_3_.


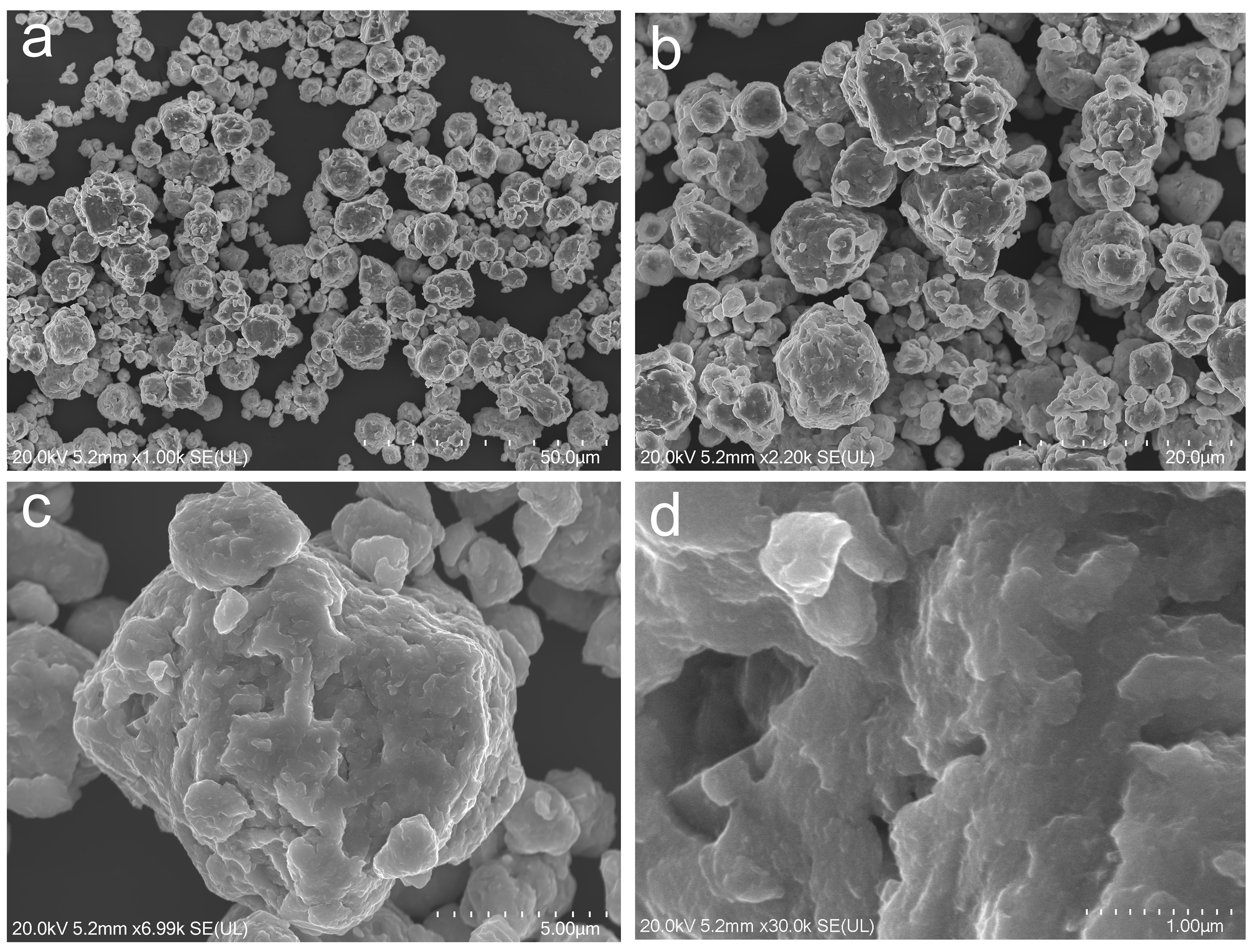


**Figure S6**. (a–d) FESEM images of the CeNi_3_-Ar sample.


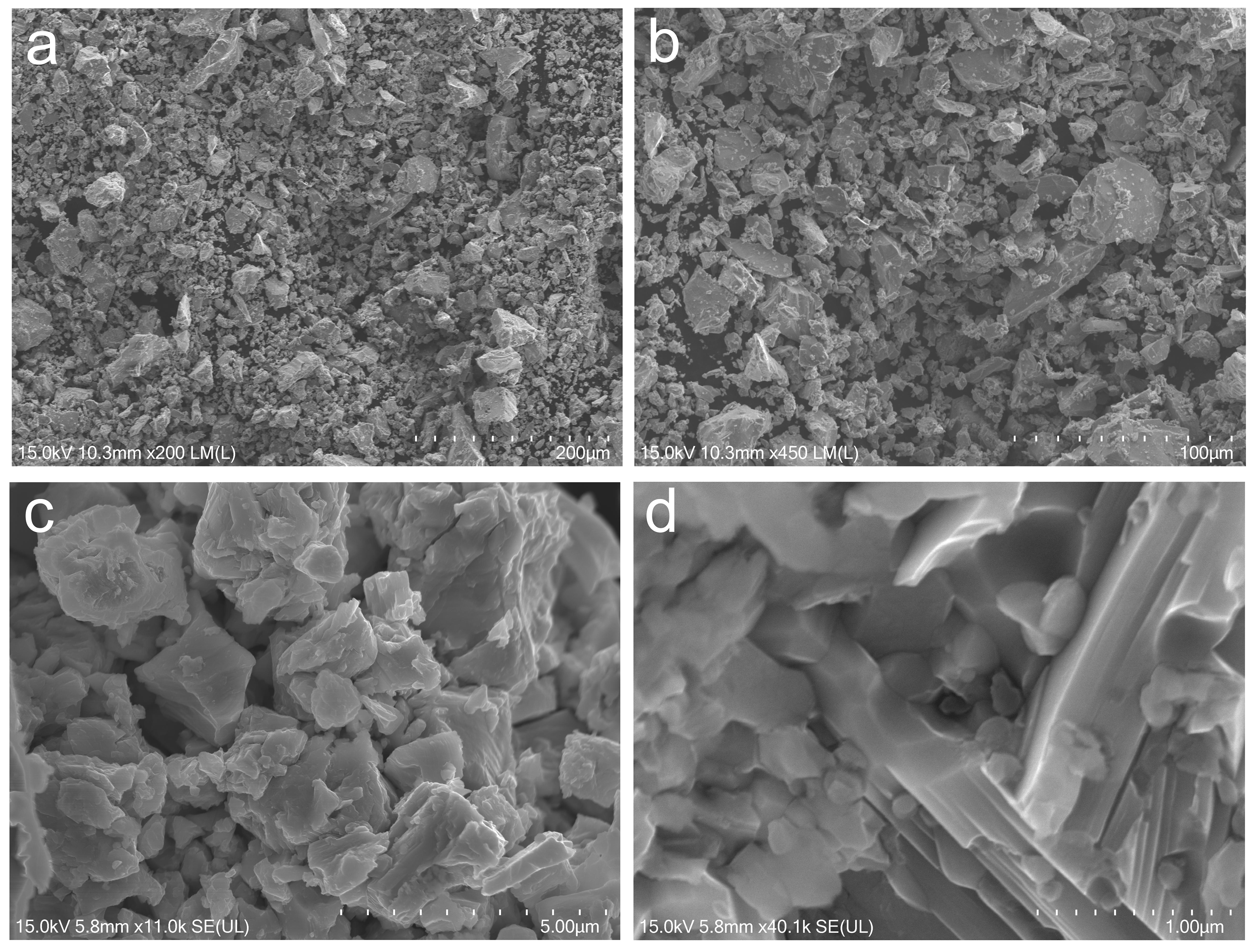


**Figure S7**. (a–d) FESEM images of the pristine CeNi_3_ sample.


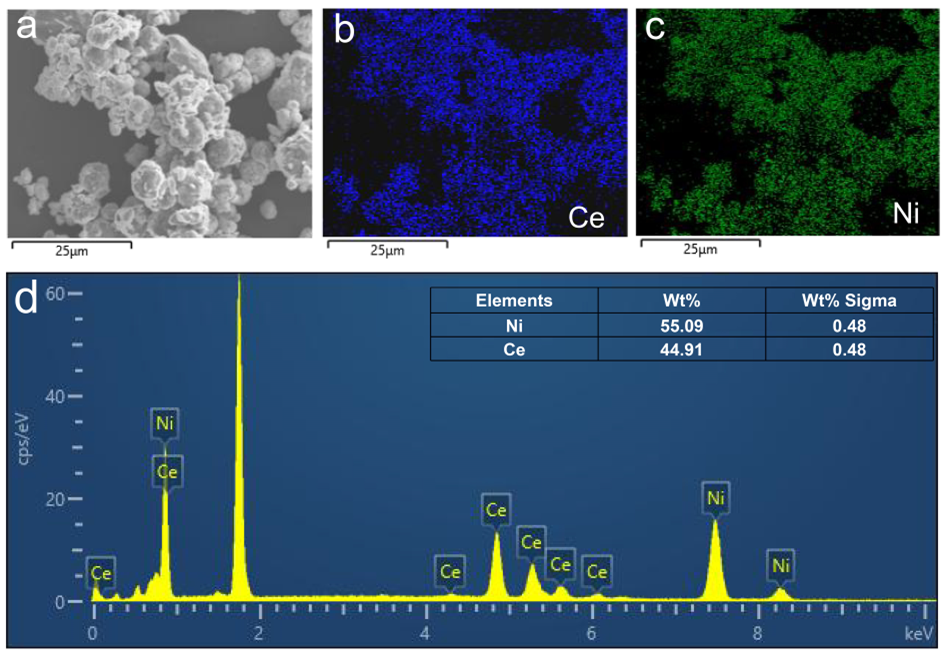


**Figure S8**. (a) FESEM image, and corresponding elemental mappings for (b) Ce species and (c) Ni species, as well as (d) EDX spectra for CeNi_3_-Ar.

**
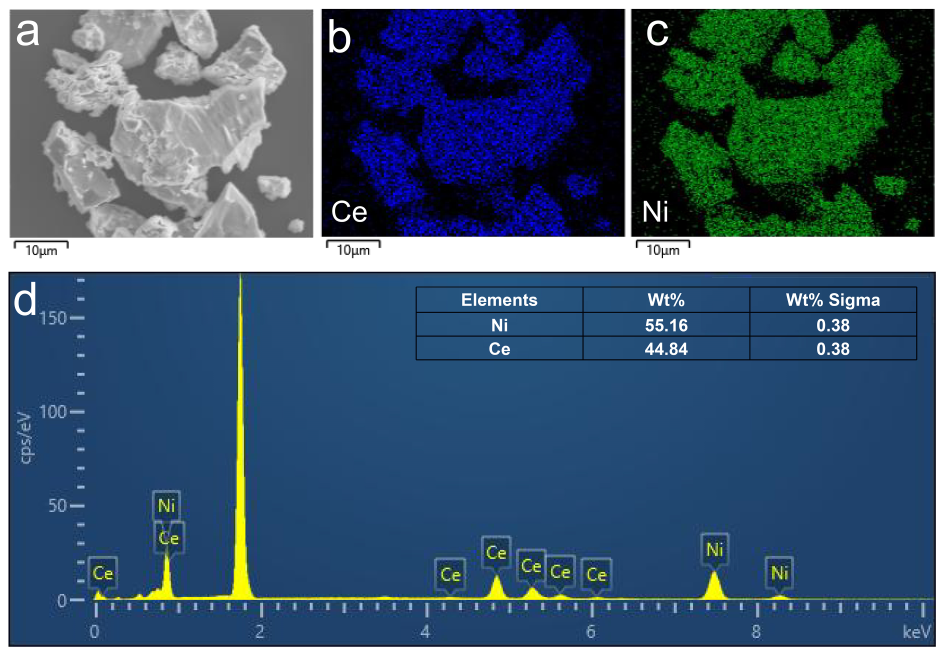
**

**Figure S9**. (a) FESEM image, and corresponding elemental mappings for (b) Ce species and (c) Ni species, as well as (d) EDX spectra for pristine CeNi_3_.


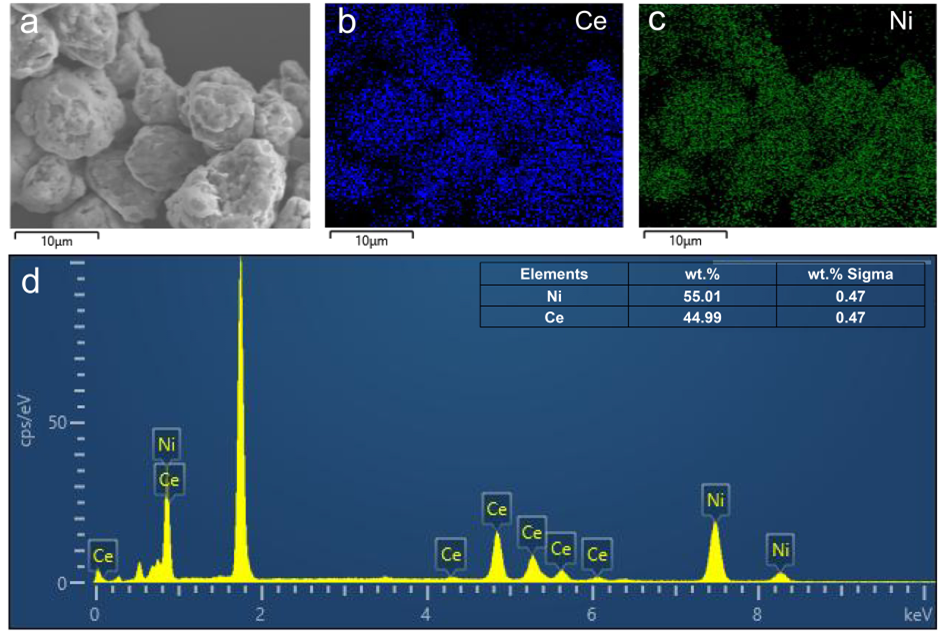


**Figure S10**. (a) FESEM image, and corresponding elemental mappings for (b) Ce species and (c) Ni species, as well as (d) EDX spectra for CeNi_3_-H.


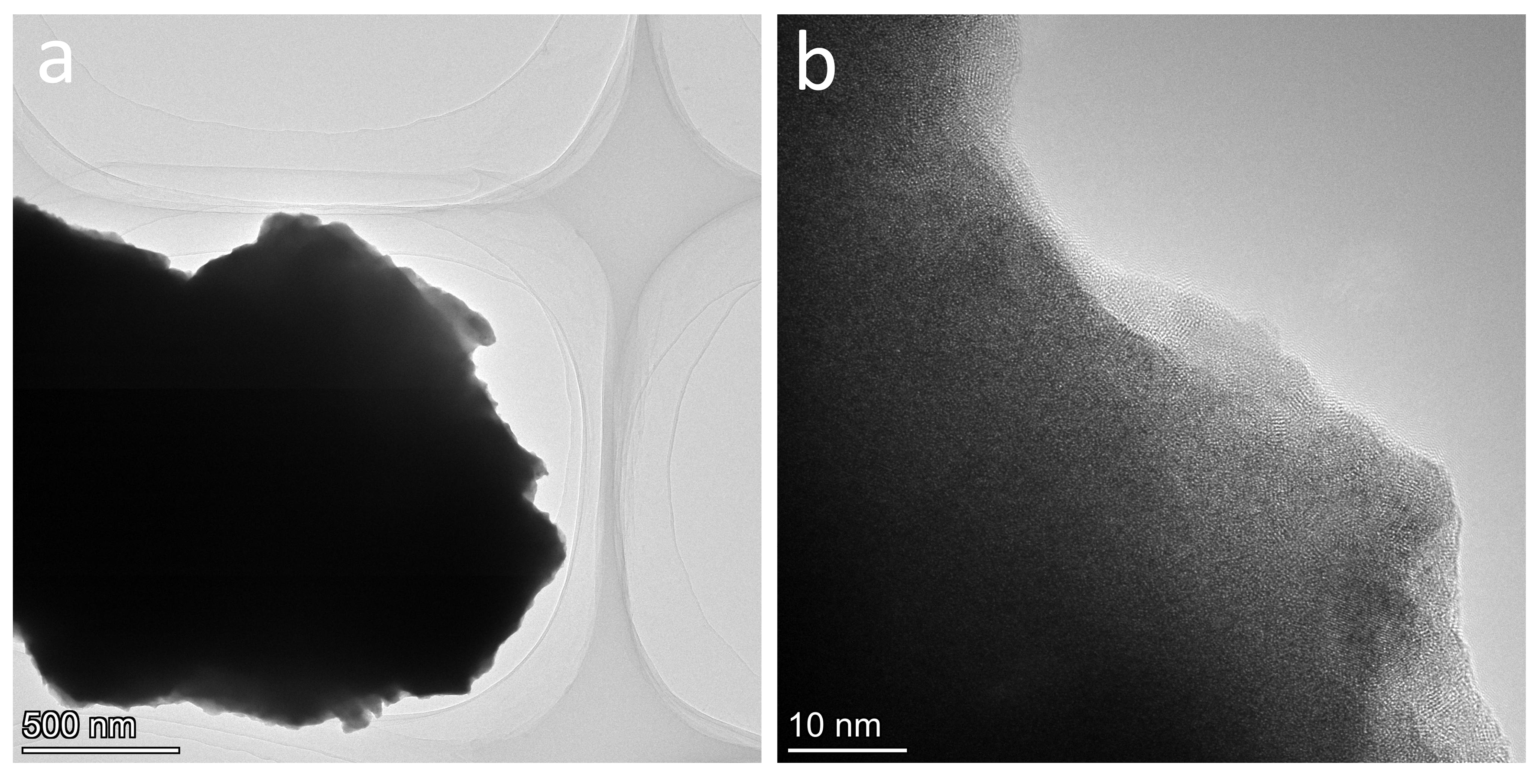


**Figure S11.** (a, b) TEM images for CeNi_3_-H.


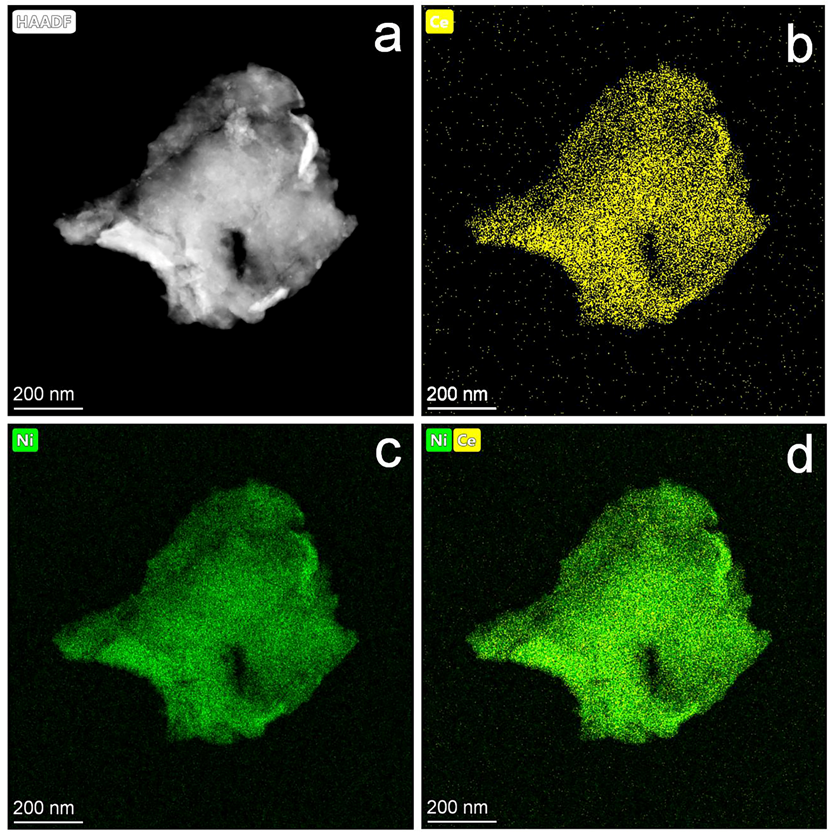


**Figure S12**. (a) HAADF image and (b–d) corresponding elemental mappings for CeNi_3_-H.


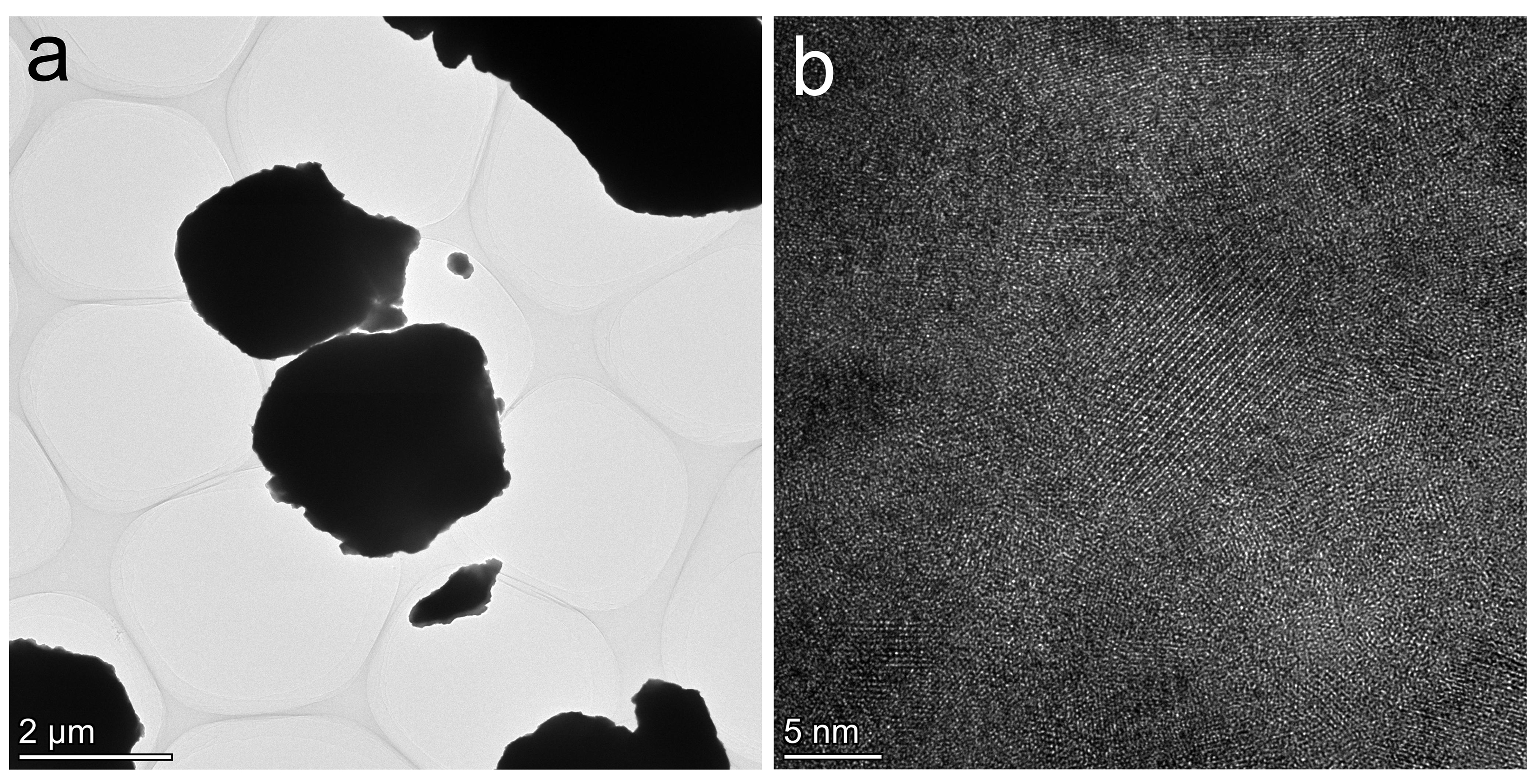


**Figure S13**. (a) TEM image and (b) corresponding HRTEM image for CeNi_3_-Ar sample.


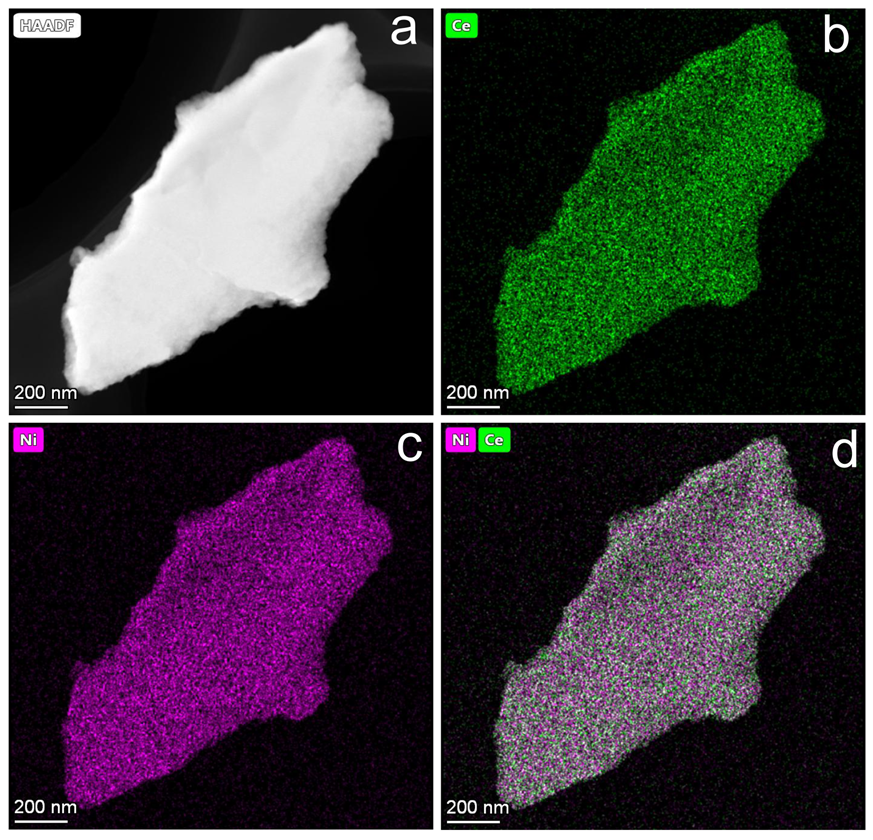


**Figure S14**. (a) HAADF image and (b–d) corresponding elemental mappings for CeNi_3_-Ar.


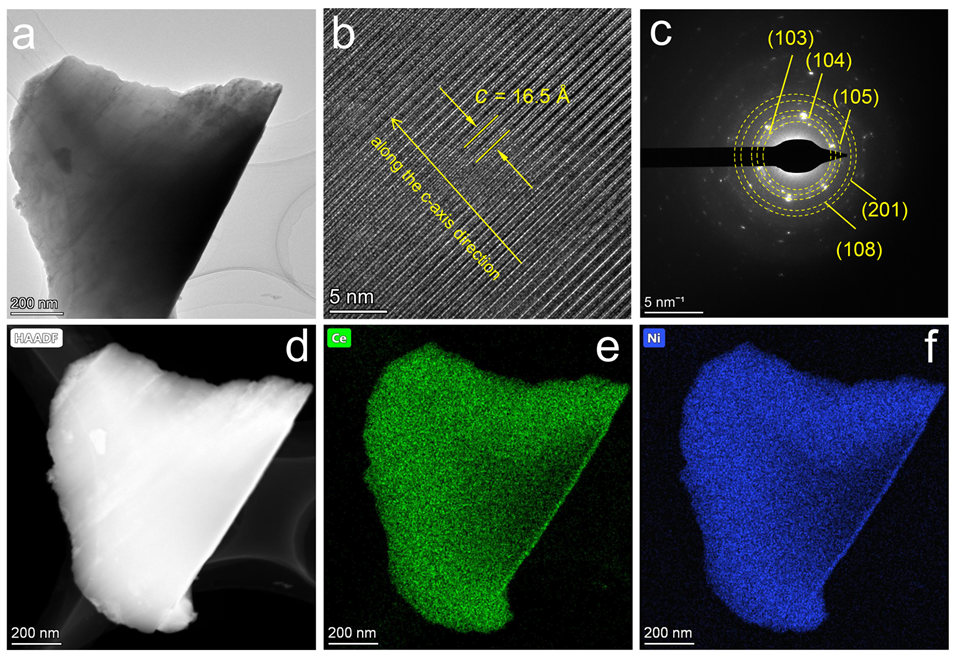


**Figure S15**. (a) TEM image, (b) HRTEM image, (c) SAED pattern, (d) HAADF image and corresponding (e, f) elemental mappings for pristine CeNi_3_.


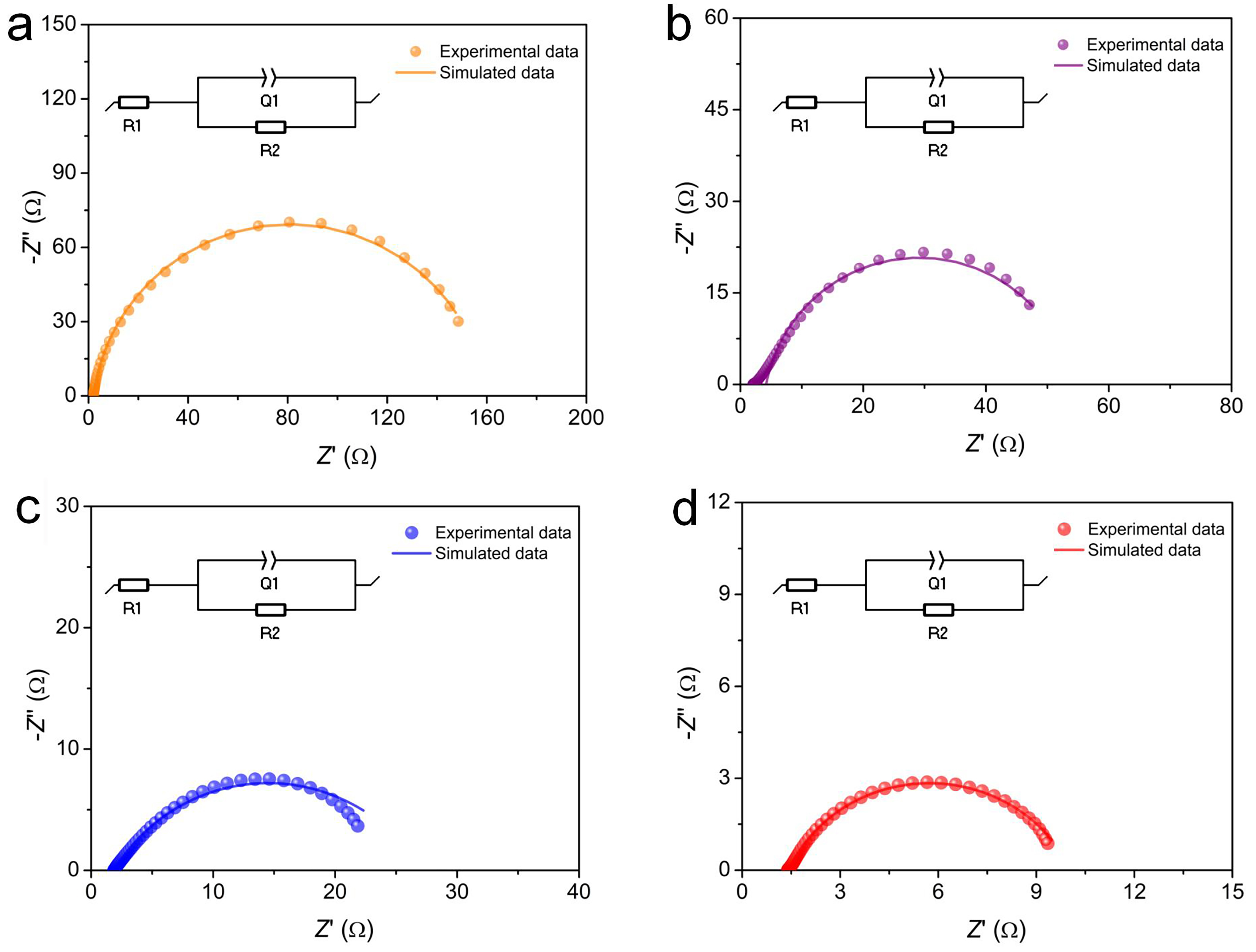


**Figure S16.** EIS fitting results for (a) CC, (b) Ni/CC, (c) CeNi_3_-Ar/CC, and (d) CeNi_3_-H electrode. The inset in the figures is the equivalent circuit for fitting.


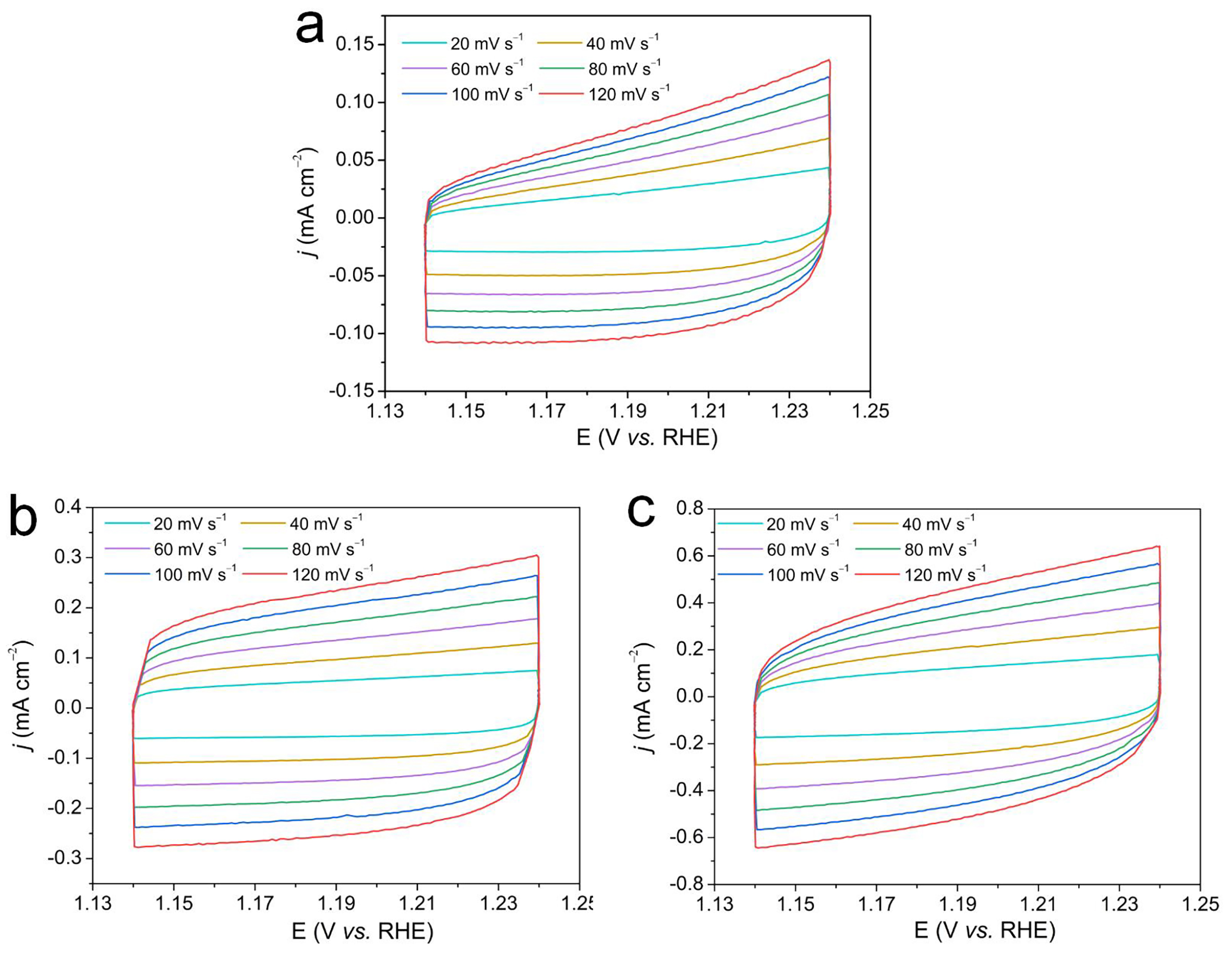


**Figure S17**. CV curves for (a) Ni/CC, (b) CeNi_3_-Ar/CC, and (c) CeNi_3_-H electrode at scan rates from 20 mV s^–1^ to 120 mV s^–1^.


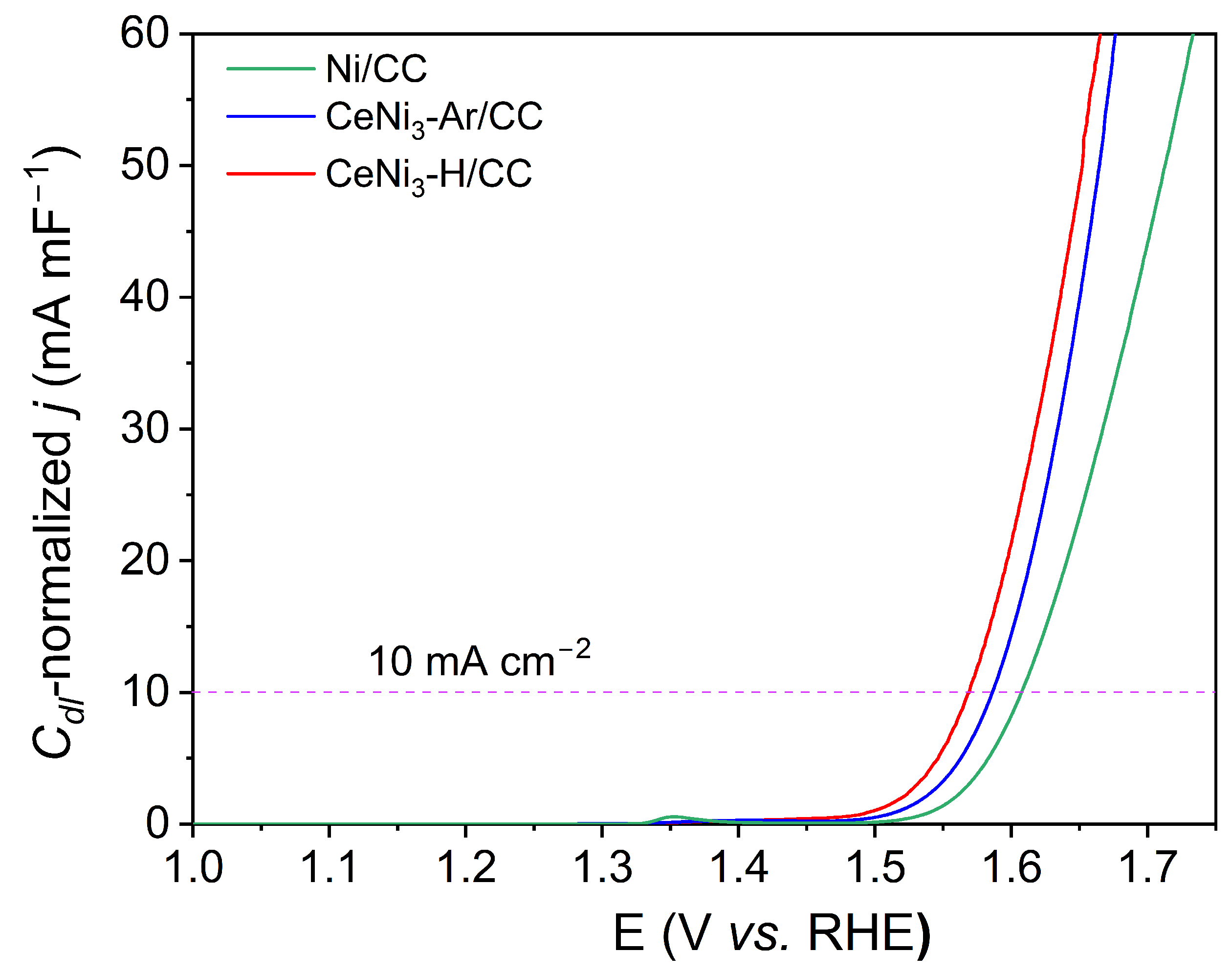


**Figure S18**. C_dl_-normalized forward CV curves for (a) Ni/CC, (b) CeNi_3_-Ar/CC, and (c) CeNi_3_-H electrode.


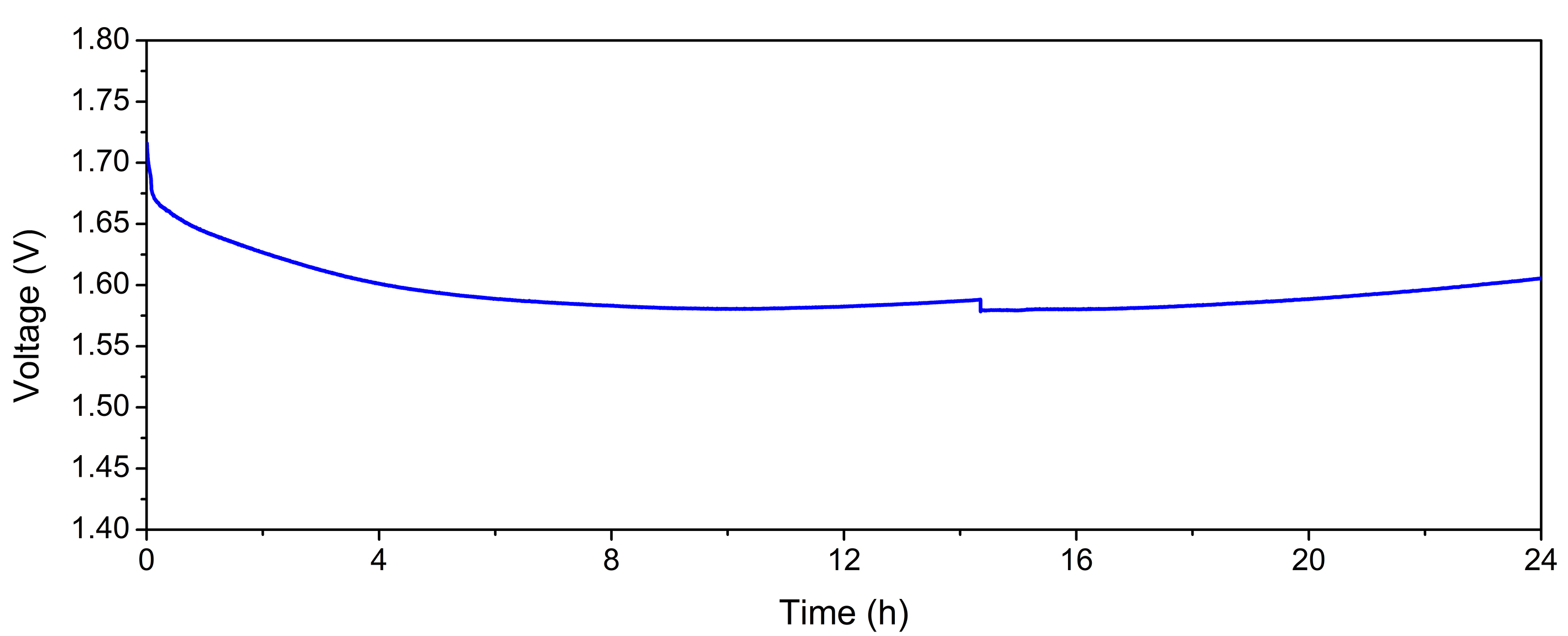


**Figure S19**. CP curve (20 mA cm^–2^) for CeNi_3_-Ar/CC electrode.


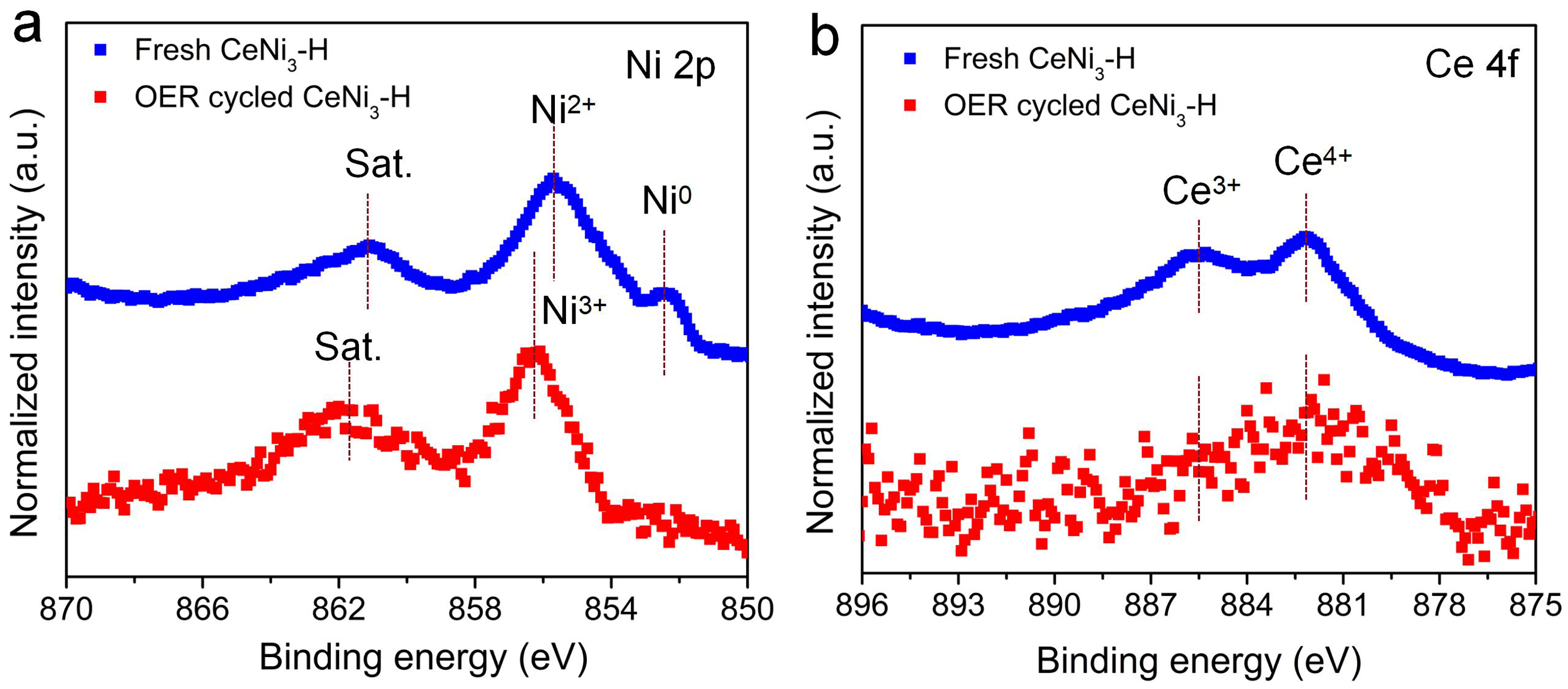


**Figure S20**. Comparison of high resolution XPS spectra for (a) Ni 2p and (b) Ce 3d in CeNi_3_/CC electrode before and after OER CP test.


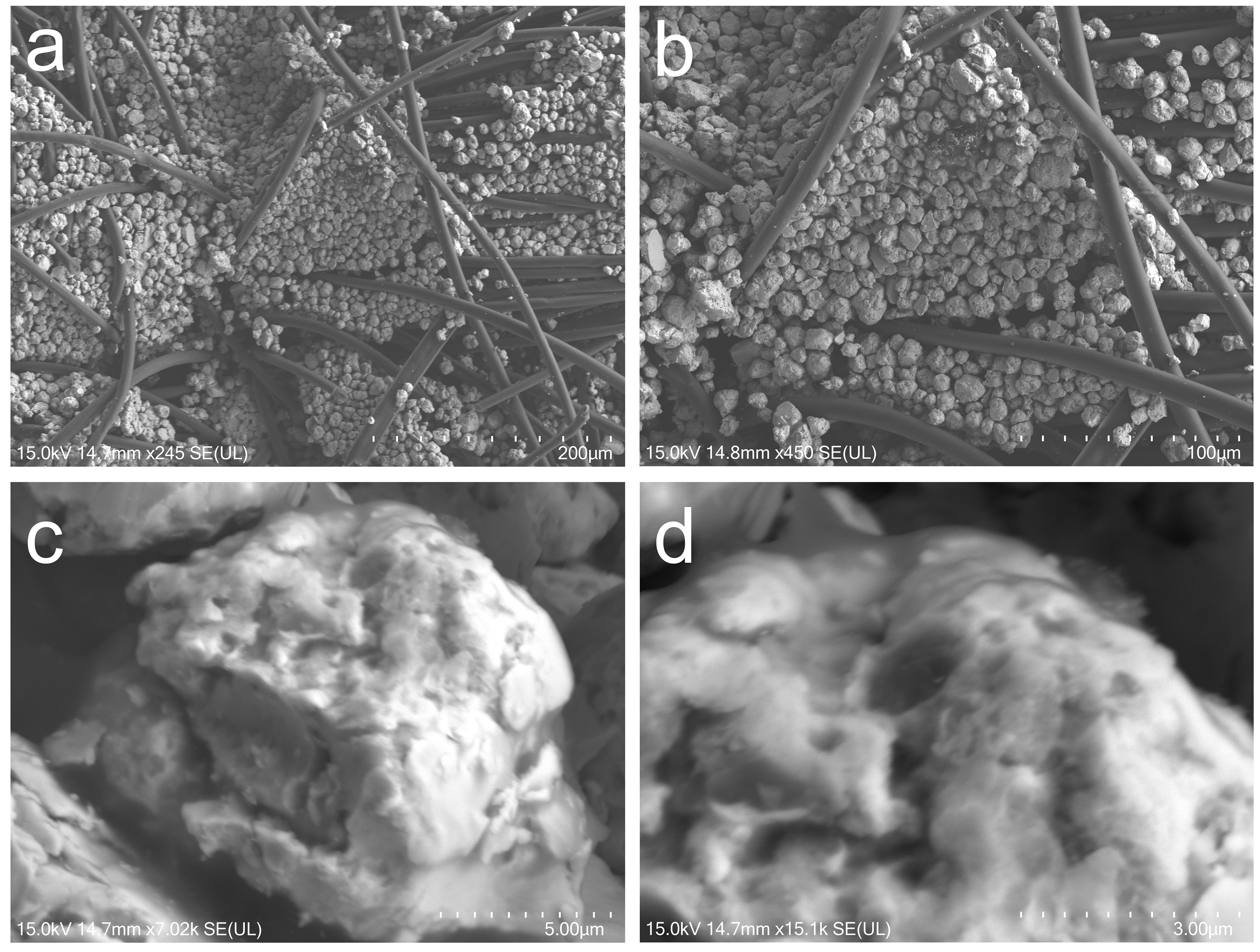


**Figure S21.** (a–d) FESEM image for CeNi_3_-H/CC electrode after OER CP test.

.


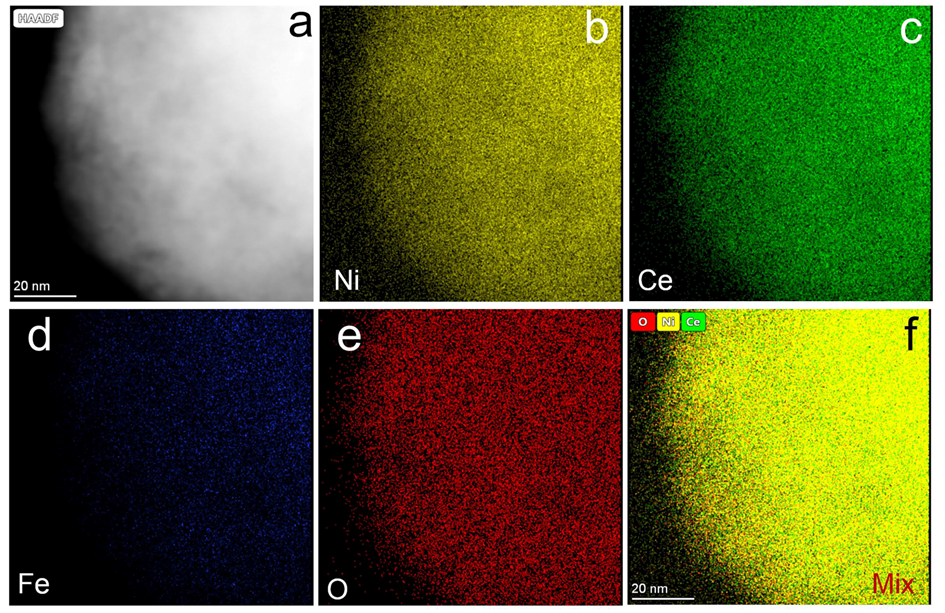


**Figure S22.** (a) HAADF image and corresponding (b–f) elemental mappings for the CeNi_3_-H particle scratched from the CeNi_3_-H/CC electrode after the OER CP test.


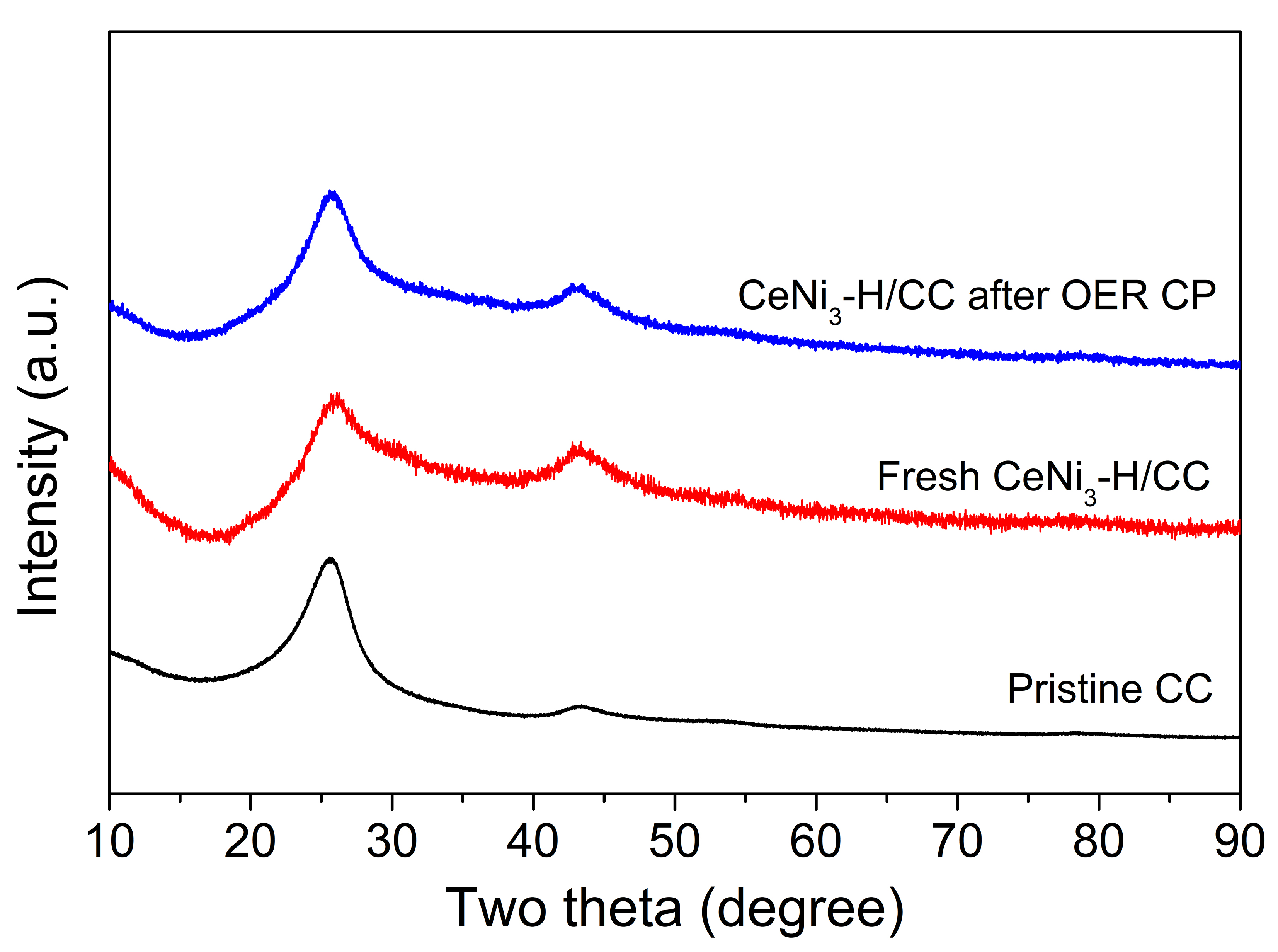


**Figure S23**. XRD patterns of CeNi_3_-H/CC electrode before and after OER CP, together with the pristine CC for comparison.


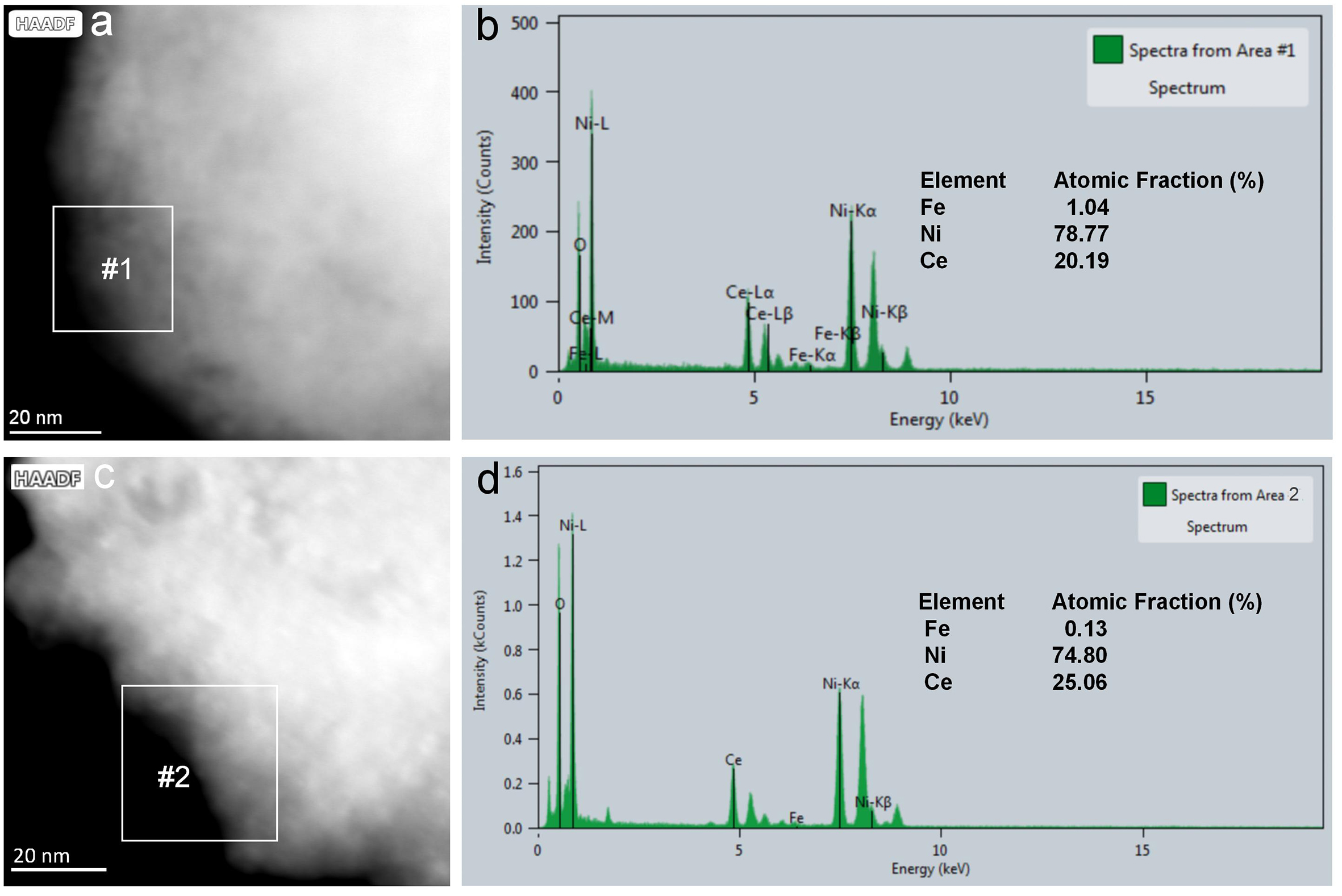


**Figure S24.** EDX results recorded from the surface edge region of the CeNi_3_-H particle scratched off from the CeNi_3_-H/CC electrode (a, b) before and (c, d) after the OER CP test.


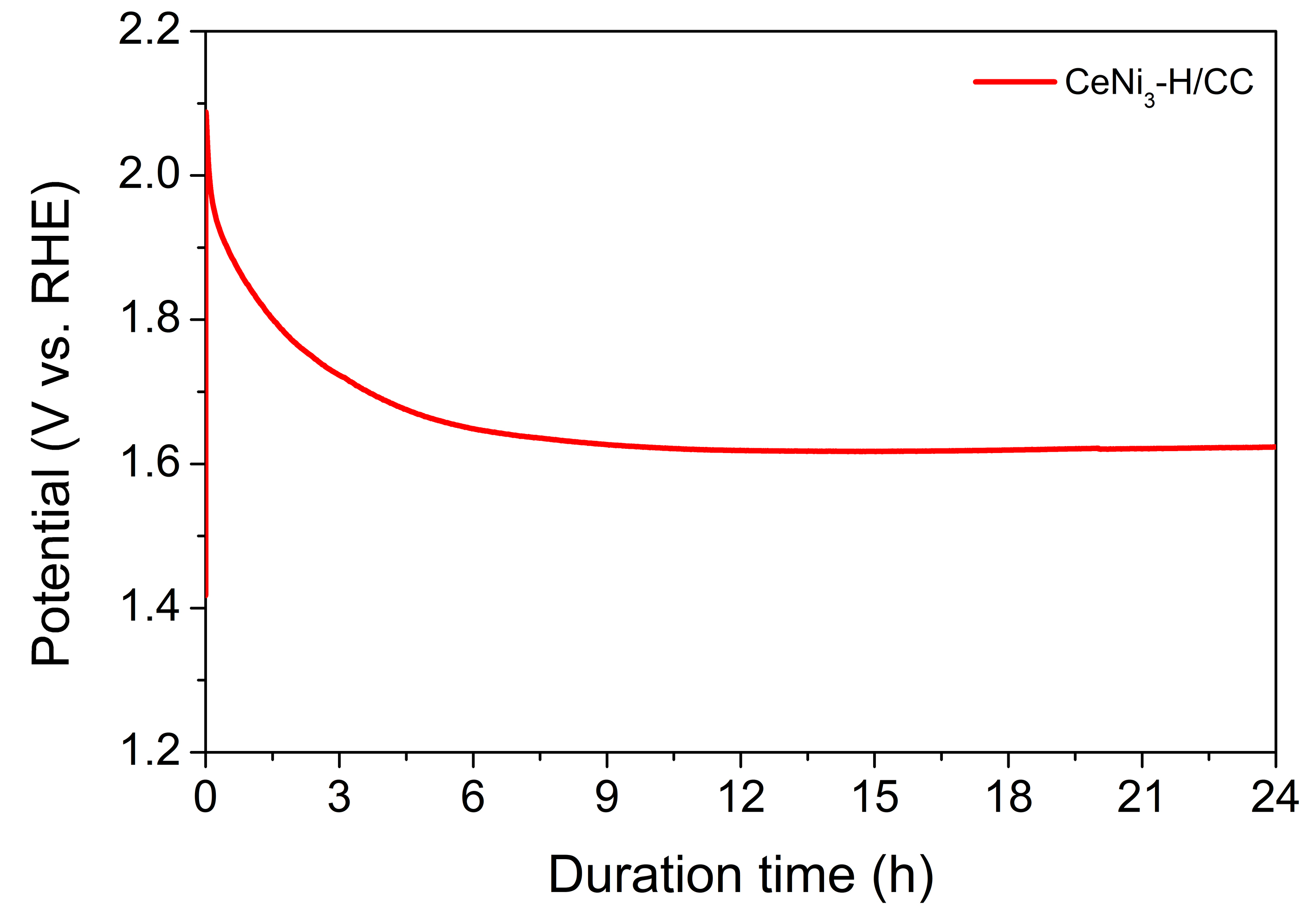


**Figure S25.** The chronopotentiometry test at 100 mA cm⁻^2^ in an H-cell for 24 h.

_
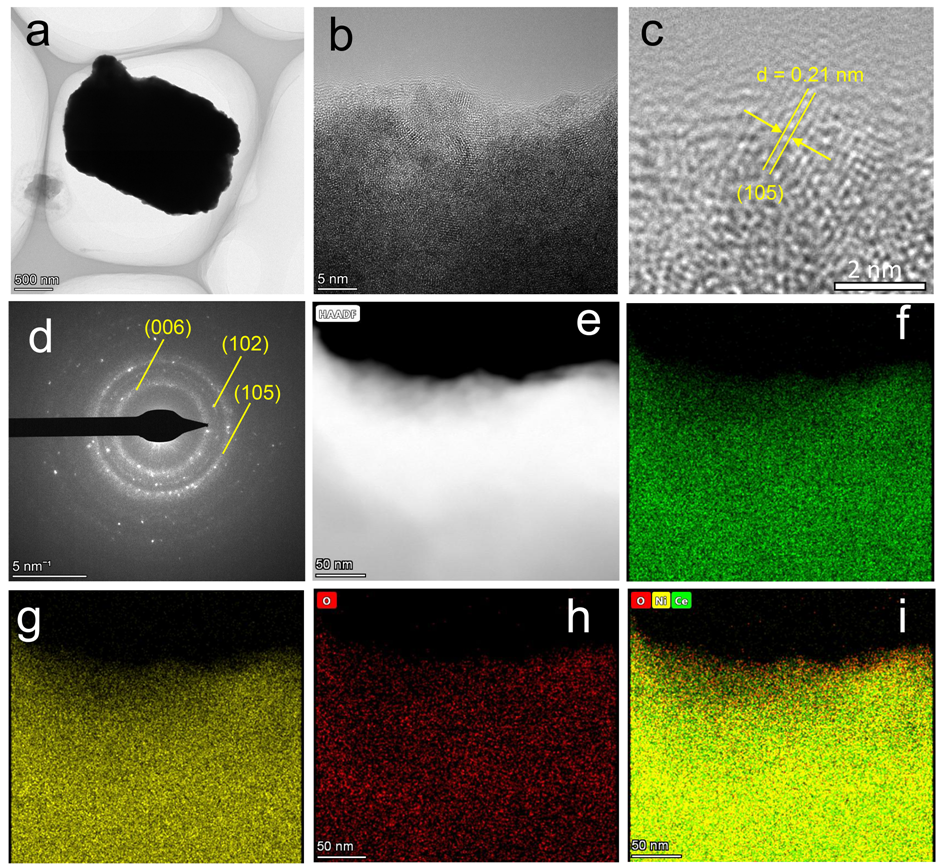
_

**Figure S26.** (a) TEM image, (b, c) HRTEM image, (d) SAED pattern, (e) HAADF image, and (f–i) corresponding elemental mappings for the CeNi_3_-Ar particle scratched off from the CeNi_3_-Ar/CC electrode after OER CP test.


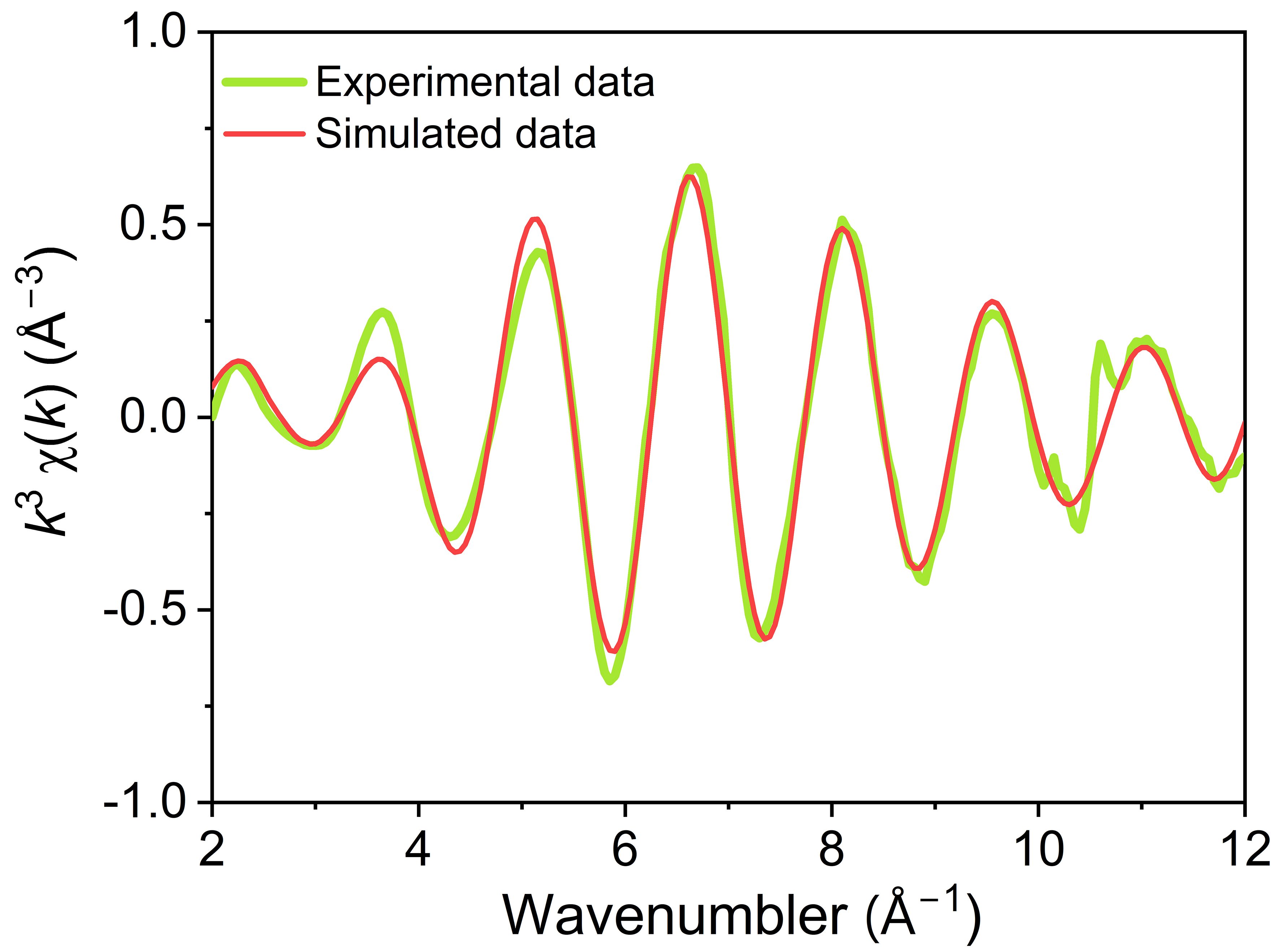


**Figure** **S27.** The *k^3^*-weighted Fourier EXAFS spectra of Ni K-edge for CeNi_3_-H/CC after OER CP.


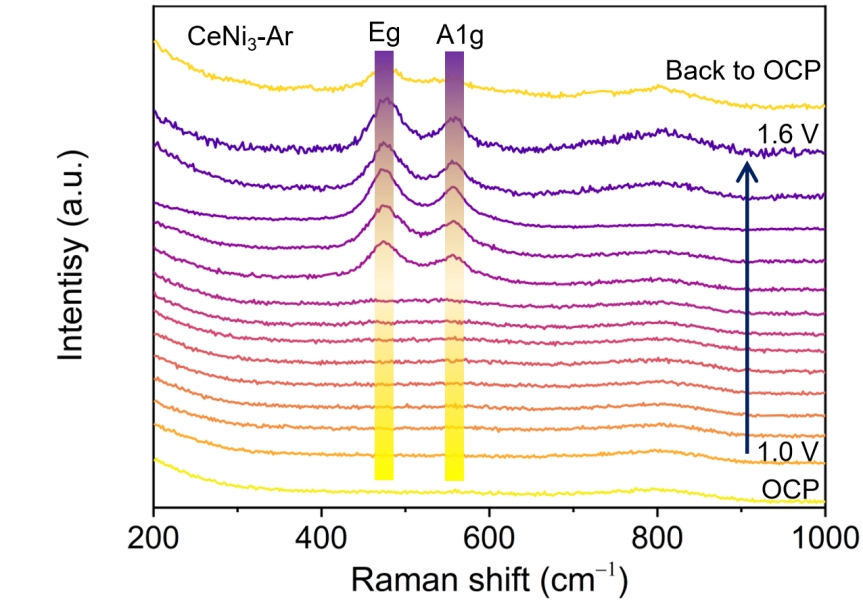


**Figure S28.** *In situ* Raman spectra for CeNi_3_-Ar, with an increased interval voltage of 50 mV from 1.0 V to 1.6 V.


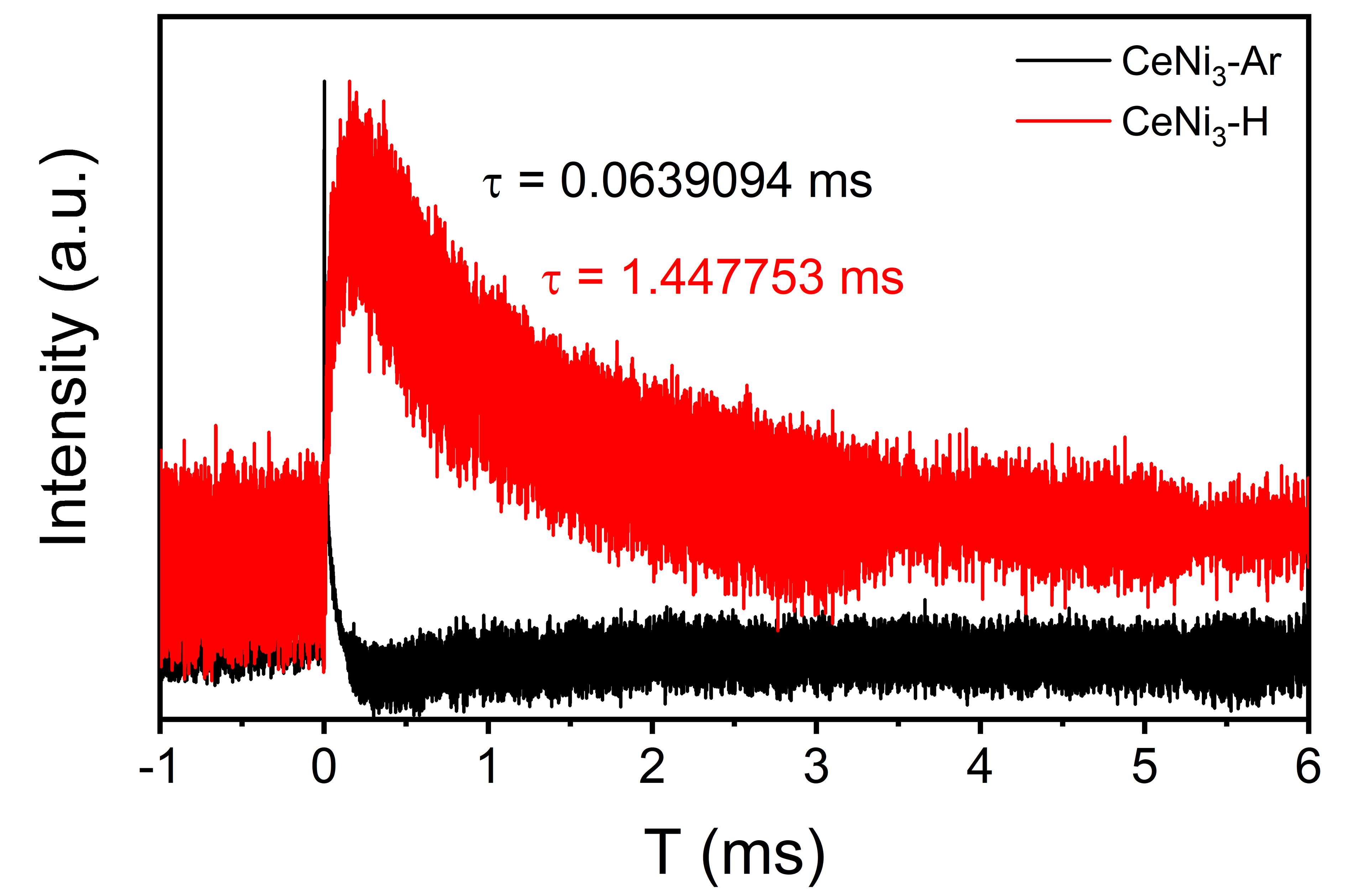


**Figure S29.** TPV curves for CeNi_3_-Ar and CeNi_3_-H powder.


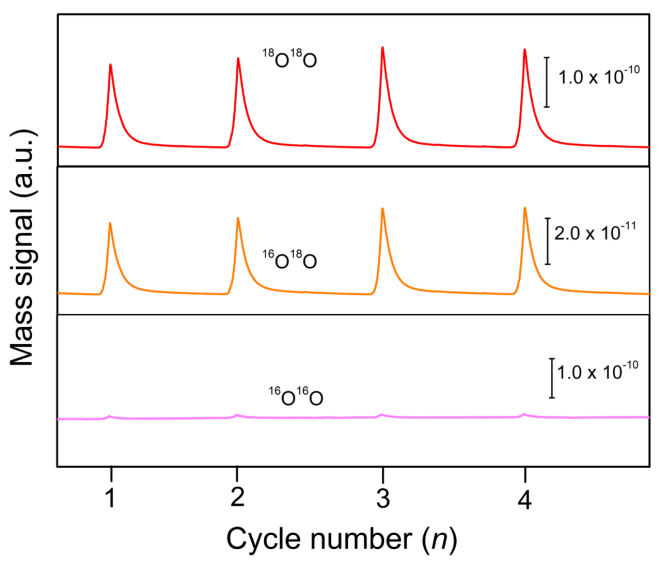


**Figure S30.** DEMS signals of ^32^O_2_ (^16^O^16^O), ^34^O_2_ (^16^O^18^O) and ^36^O^2^ (^18^O^18^O) in ^18^O isotope labeling process of activated CeNi_3_-H in H_2_^18^O aqueous potassium hydroxide solution electrolyte.

**
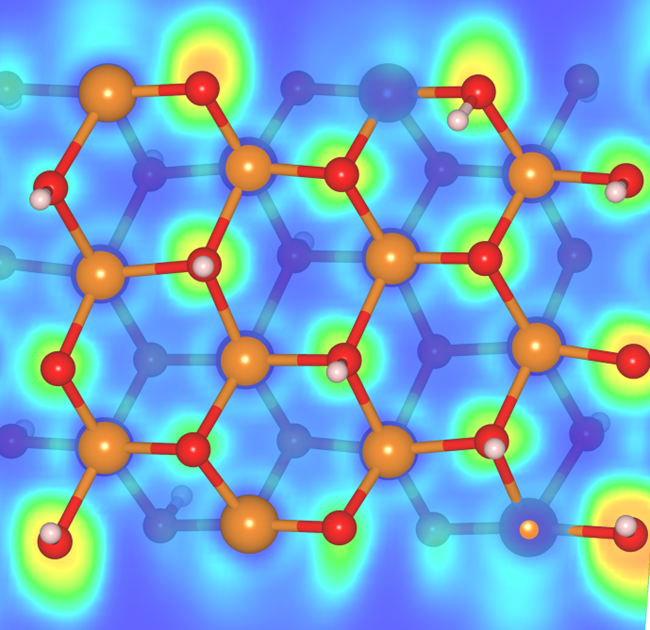
**

**Figure S31.** Electron local functional map for the (012) facet of NiOOH.

**
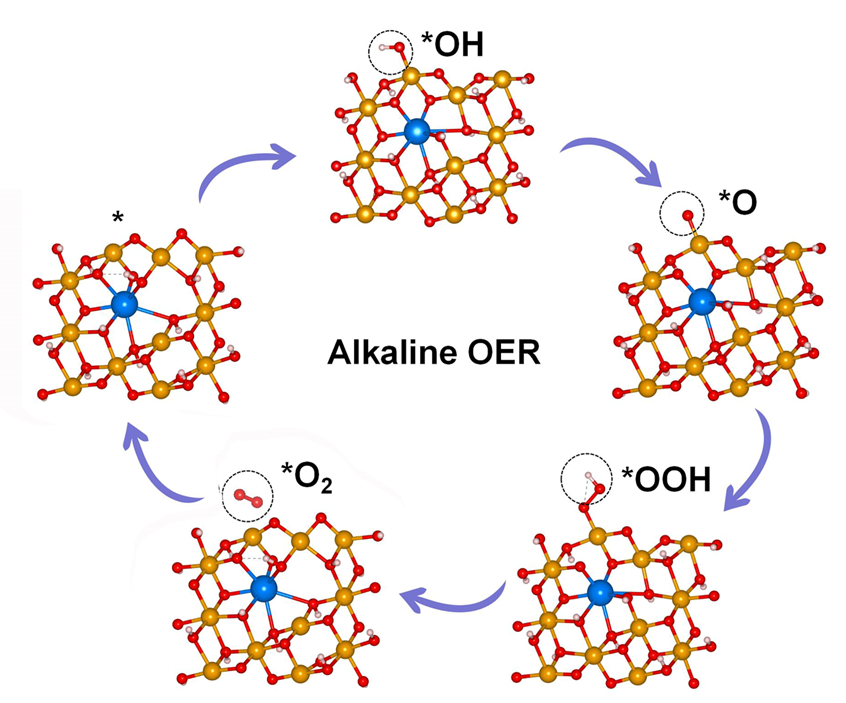
**

**Figure S32.** The proposed OER pathways for the Ce-NiOOH model.


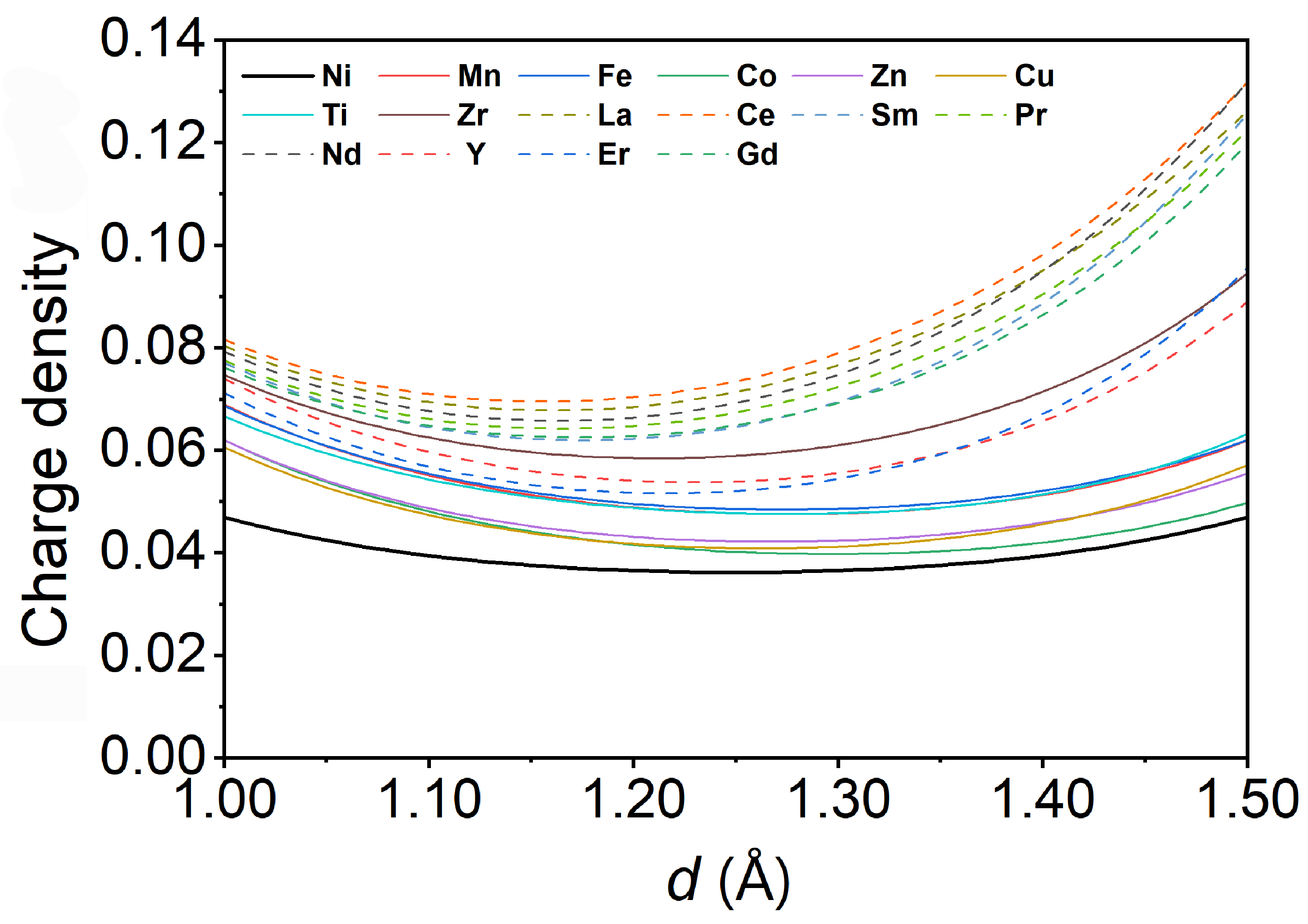


**Figure S33.** Charge-density profiles along the internuclear axis (from 1 to 1.5 Å, especially in the midpoint region).


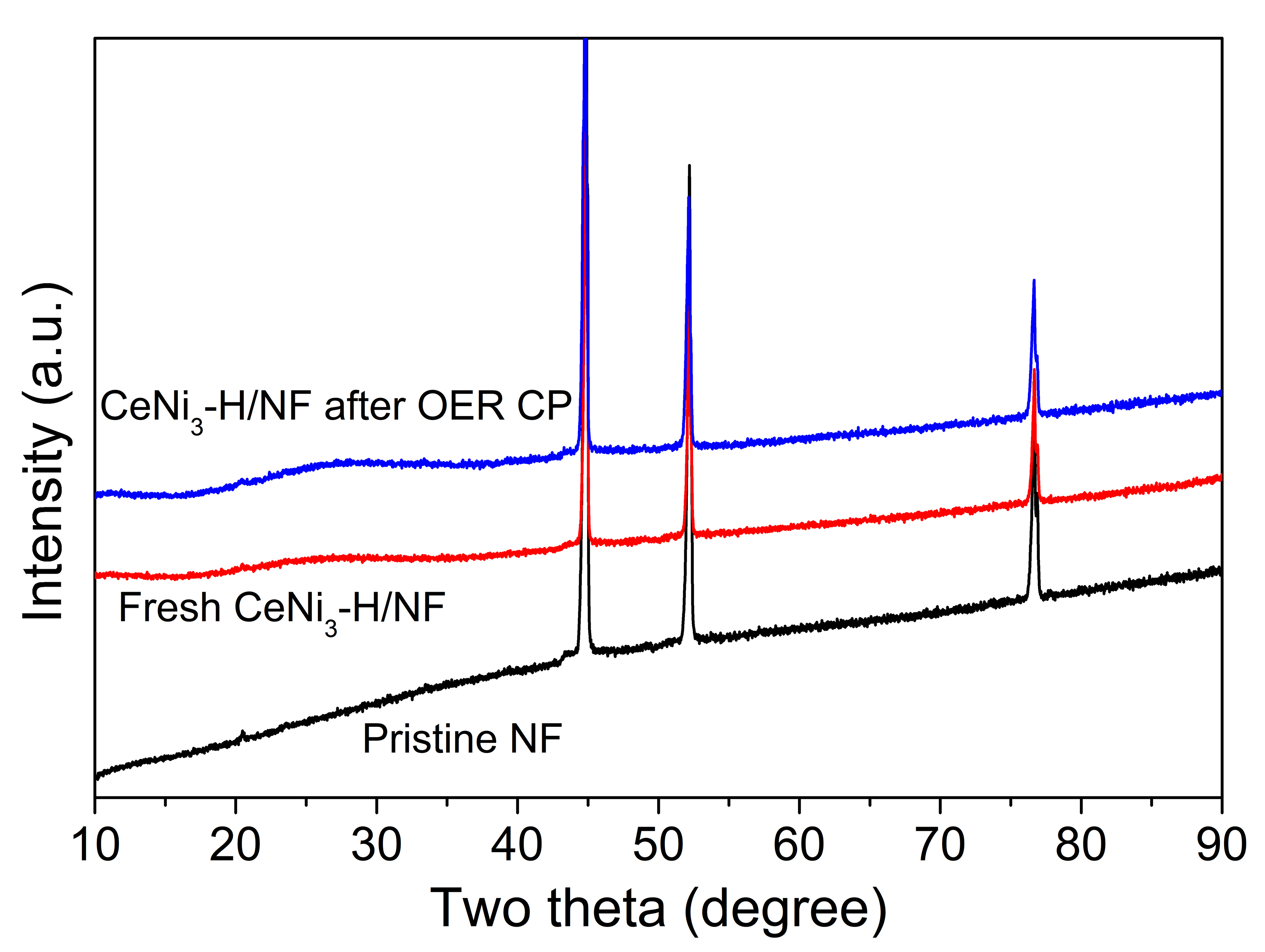


**Figure S34.** XRD patterns of CeNi_3_-H/NF before and after OER CP test, together with the pristine NF for comparison.

**
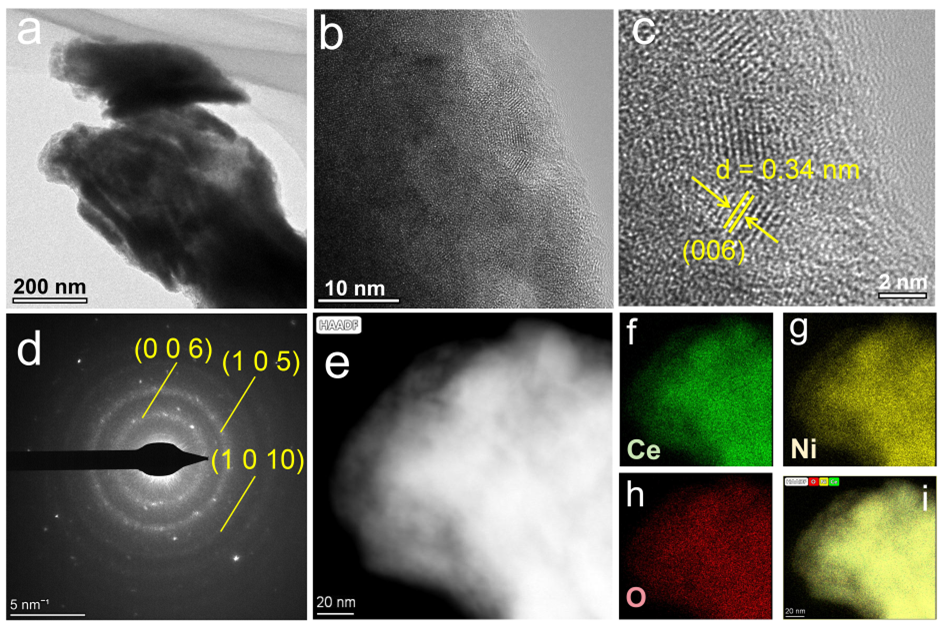
**

**Figure S35.** (a) TEM image, (b, c) HRTEM image, (d) SAED pattern, (e) HAADF image and (f–i) corresponding elemental mappings for the CeNi_3_-H particle scratched from the CeNi_3_-H/CC electrode after OER CP test.


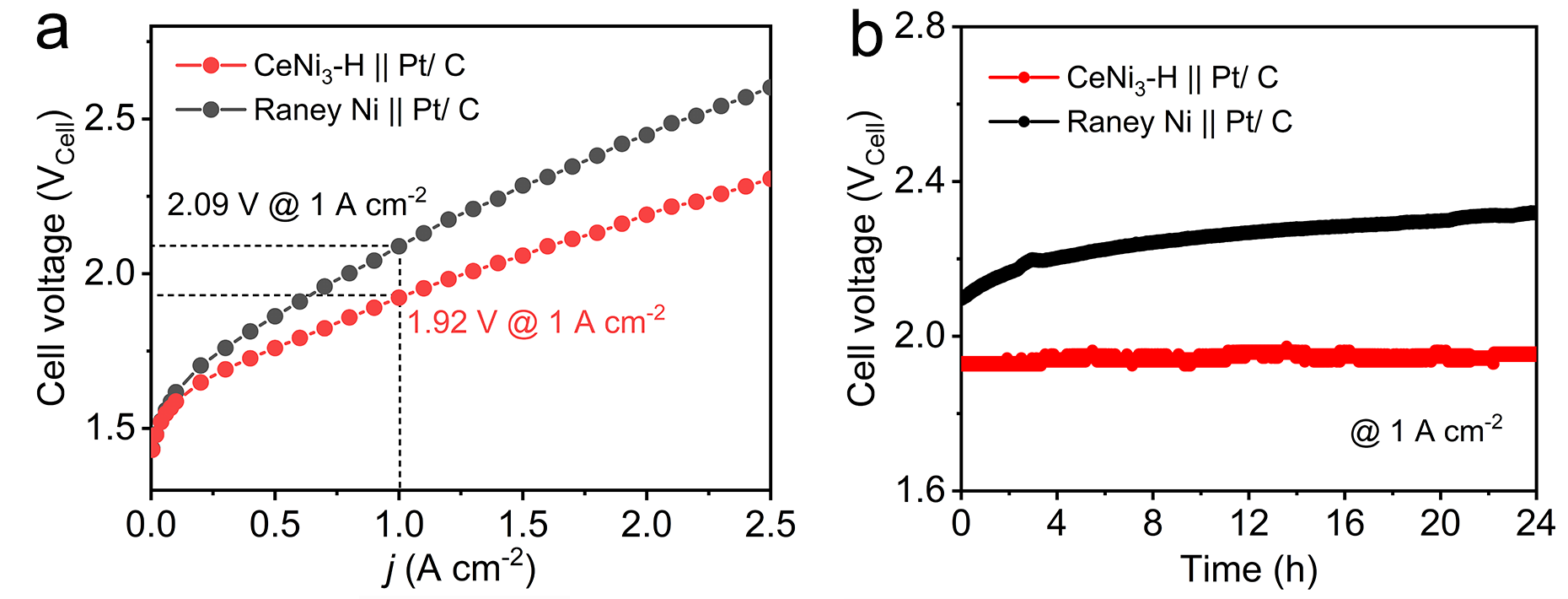


**Figure S36.** (a) Polarization curves for the AEMWE with various electrodes at 60 °C; (b) The stability at 1.0 A cm^−2^ for Pt/C(−)||CeNi_3_-H(+) AEMWE.

**TABLES**

**Table S1.** Structural parameters for pristine CeNi_3_ and CeNi_3_-Ar compounds refined from the experimental XRD profiles.

| Sample | Space group | Lattice parameters (Å) | | | Abundance (wt.%) |
| --- | --- | --- | --- | --- | --- |
|  |  | *a* | *b* | *c* |  |
| Pristine CeNi_3_ | *P*6_3_*/mmc* | 4.9681(4) | 4.9681(4) | 16.533(1) | 100 |
| CeNi_3_-Ar | *P*6_3_*/mmc* | 4.956(6) | 4.956(6) | 16.56(3) | 100 |

**Table S2.** EXAFS fitting parameters at the Ni K-edge for various samples(*Ѕ*_0_^2^=0.70).

| Sample | Shell | *CN^a^* | *R*(Å)*^b^* | *σ*^2^(Å^2^)*^c^* | Δ*E*_0_(eV)*^d^* | *R* factor |
| --- | --- | --- | --- | --- | --- | --- |
| Pristine CeNi_3_ | Ni-Ni | 5.2±0.1 | 2.48±0.01 | 0.0065 | 5.9 | 0.007 |
|  | Ni-Ce | 0.8±0.1 | 2.95±0.01 | 0.0065 | 5.9 |  |
| CeNi_3_-Ar | Ni-Ni | 4.1±0.1 | 2.46±0.01 | 0.0061 | 3.5 | 0.006 |
|  | Ni-Ce | 0.4±0.1 | 2.82±0.01 | 0.0061 | 3.1 |  |
| CeNi_3_-Ar | Ni-Ni | 3.6±0.1 | 2.44±0.01 | 0.0089 | 1.6 | 0.006 |
|  | Ni-Ce | 0.1±0.1 | 2.82±0.01 | 0.0089 | 4.5 |  |
| CeNi_3_-H Post OER CP | Ni-Ni | 3.4±0.1 | 2.43±0.01 | 0.0079 | 2.0 | 0.005 |
|  | Ni-O | 0.2±0.1 | 1.94±0.01 | 0.0079 | 2.0 |  |

*^a^CN*, coordination number; *^b^R*, distance between absorber and backscatter atoms; *^c^σ*^2^, Debye-Waller factor to account for both thermal and structural disorders; *^d^ΔE*_0_, inner potential correction; *R* factor indicates the goodness of the fit. *S*_0_^2^ was fixed to 0.70, according to the experimental EXAFS fit of Ni-foil by fixing CN as the known crystallographic value. Fitting range: 3.0 ≤ *k* (Å^-1^) ≤ 12.0 and 1.0 ≤ *R* (Å) ≤ 3.5 (Ni-foil); 3.0 ≤ *k* (Å^-1^) ≤ 12.0 and 1.0 ≤ *R* (Å) ≤ ~3.0 (Sample-Ni、NiO). A reasonable range of EXAFS fitting parameters: 0.700 < *Ѕ*_0_^2^ < 1.000; *CN >* 0; *σ*^2^ > 0 Å^2^; Δ*E*_0_ < 10 eV; *R* factor < 0.02.

**Table S3.** *R*_ct_ (Ω), *R*_s_ (Ω), CPE (*F* x *s*^(^*^a2^*^-1)^) and *a^2^* for CC, Ni/CC, CeNi_3_-Ar/CC and CeNi_3_-H/CC obtained from EIS fitting at 1.64 V (*vs.* RHE).

| **Sample** | ***R*_s_ (Ω)** | ***R*_ct_ (Ω)** | ***CPE*** | ***a^2^*** |
| --- | --- | --- | --- | --- |
| CC | 1.952 | 158.1 | 0.00081 | 0.9168 |
| Ni/CC | 4.2 | 49.88 | 0.00415 | 0.8843 |
| CeNi_3_-Ar/CC | 1.964 | 25.3 | 0.00485 | 0.6682 |
| CeNi_3_-H/CC | 1.422 | 8.617 | 0.00481 | 0.7423 |

**Table S4.** ICP-MS analysis of the electrolyte after 100 h chronopotentiometry with CeNi_3_-H catalyst.

| **Sample** | **Ce (μg L^-1^)** | **Ni (μg L^-1^)** |
| --- | --- | --- |
| CeNi_3_-H/CC | 8.21 | 2.66 |

**Table S5.** The relative polarizability (RP) between metal and Ni metal analyzed by DFT.

| d-block metal | Ni | Cu | Zn | Co | Fe | Mn | Ti | Zr |
| --- | --- | --- | --- | --- | --- | --- | --- | --- |
| RP | 0.00 | 0.04 | 0.07 | 0.13 | 0.36 | 0.50 | 0.58 | 0.64 |
| f-block metal | Y | Sm | Nd | Er | La | Gd | Ce | Pr |
| RP | 0.66 | 0.73 | 0.82 | 1.37 | 1.42 | 1.53 | 1.65 | 1.81 |

**Table S6.** The comparison of OER activity and stability of CeNi_3_-H/NF with those of recently reported NF-supported intermetallics-, Ni-based and NiOOH OER catalysts in alkaline solution.

| **Electrode** | **Electrolyte** | **Overpotential @ 500 mA cm^–2^** | | **Stability**  ***j* (mA cm^–2^) @ *t* (h)** | **Ref.** |
| --- | --- | --- | --- | --- | --- |
| MoO*_x_*/Ni_3_S_2_/NF | 1.0 M KOH | 496 mV | 15@200 | | [S14] |
| MnO_x_/NiFeP/NF | 1.0 M KOH | 296 mV | 500@120 | | [S15] |
| Ni-FeO*_x_*/FeNi_3_/NF | 1.0 M KOH | 345 mV | 50@200 | | [S16] |
| Fe–Co PBA@Ni(OH)_2_/NF | 1.0 M KOH | 374 mV | 625@40 | | [S17] |
| Ni_3_Fe–FeV_2_O_4_@C/NF | 1.0 M KOH | 290 mV | 500@50 | | [S18] |
| NiPd@NF_120_ | 1.0 M KOH | 310 mV | 20@24 | | [S19] |
| Ni_0.65_Ga_0.30_Fe_0.05_/NF | 1.0 M KOH | 410 mV | 20@100 | | [S20] |
| Fe/Ni/Mn_0.4_-MIL-53/NF | 1.0 M KOH | 290 mV | 30@60 | | [S21] |
| FeNi(MoO_4_)*_x_*/NF | 1.0 M KOH | 320 mV | 1000@90 | | [S22] |
| NiFeMo-N2/NF | 1.0 M KOH | 300 mV | 10@100 | | [S23] |
| NiFe-LDH/NF-S-3h | 1.0 M KOH | 309 mV | 500@150 | | [S24] |
| NiSe/NF | 1.0 M KOH | 730 mV | 100@12 | | [S25] |
| NiCoP/NF | 1.0 M KOH | 365 mV | 500@24 | | [S26] |
| FeCoNiMnW/NF | 1.0 M KOH | 355 mV | 500@1200 | | [S27] |
| Ni-Fe-OH@Ni_3_S_2_/NF | 1.0 M KOH | 370 mV | 500@50 | | [S28] |
| Zn-NiOOH | 1.0 M KOH | N.A. | 500@200 | | [S29] |
| NiFe-2.0-LDH | 1.0 M KOH | 278 mV | 1000@80 | | [S30] |
| NiFeCoMnAl | 1.0 M KOH | N.A. | 50@10 | | [S31] |
| β-NiOOH@FeOOH | 1.0 M KOH | N.A. | 100-250-500@150 | | [S32] |
| a-NiOOH/CoOOH | 1.0 M KOH | N.A. | 10-50-100@200 | | [S33] |
| FeOOH@NiOOH/NF | 1.0 M KOH | N.A. | 20@24 | | [S34] |
| CeNi_3_-H/NF | 1.0 M KOH | 389mV | ~600@200 | | **This work** |

**References**

[S1] F. Izumi, T. Ikeda. *Mater. Sci. Forum.*, 2000, **321-324**, 198.

[S2] K. Momma, F. Izumi. *J. Appl. Crystallog*r., 2011, **44**, 1272.

[S3] G. Kresse, J. Furthmüller. *Phys. Rev. B*., 1996, **54,** 11169.

[S4] G. Kresse, J. Furthmüller. *Comput. Mater. Sci*., 1996, **6**, 15.

[S5] P. E. Blöchl. *Phys. Rev. B*., 1994, **50**, 17953.

[S6] J. P. Perdew, K. Burke, M. Ernzerhof. *Phys. Rev. Lett*., 1996, **77**, 3865.

[S7] J. Wang, Y. Gao, H. Kong, J. Kim, S. Choi, F. Ciucci, Y. Hao, S. H. Yang, Z. P. Shao, J. Lim. *Chem. Soc. Rev*., 2020, **49**, 9154.

[S8] M. Valiev, E. J. Bylaska, N. Govind, K. Kowalski, T. P. Straatsma, H. J. Van Dam, D. Wang, J. Nieplocha, E. Apra, T. L. Windus.*Comput. Phys. Commun.* **2010,** *181*, 1477.

[S9] C. Adamo, V. Barone. *J. Chem. Phys.* **1999,** *110*, 6158.

[S10] F. Weigend, R. Ahlrichs. *Phys. Chem. Chem. Phys.* **2005,** *7*, 3297.

[S11] R. Gulde, P. Pollak, F. Weigend. *J. Chem. Theory Comput.* **2012,** *8*, 4062.

[S12] D. Andrae, U. Haeussermann, M. Dolg, H. Stoll, H. Preuss, *Theor. Chim. Acta* **1990,** *77*, 123.

[S13] M. Dolg, H. Stoll, A. Savin, H. Preuss, *Theor. Chim. Acta* **1989,** *75*, 173.

[S14] Y. Y. Wu, G. D. Li, Y. P. Liu, L. Yang, X. R. Lian, T. Asefa, X. X. Zou. *Adv. Funct. Mater*., 2016, **26**, 4839.

[S15] P. Wang, Y. Z. Luo, G. X. Zhang, M. J. Wu, Z. S. Chen, S. H. Sun, Z. C. Shi. *Small,* 2022, **18**, 2105803.

[S16] A. Qayum, X. Peng, J. F. Yuan, Y. D. Qu, J. H. Zhou, Z. L. Huang, H. Xia, Z. Liu, D. Q. Tan, P. K. Chu, F. Lu, L. S. Hu. *ACS Appl. Mater.,* 2022, **14**, 27842.

[S17] Z. S. Wang, S. H. Jiao, B. R. Wang, Y. T. Kang, W. Yin, X. Y. Lv, Q. Zhang, Z. W. Zhang, Y. Chen, G. S. Pang. *Int. J. Hydrogen. Energy* 2021, **46**, 8345.

[S18] H. Zhang, G. F. Qian, T. Q. Yu, J. L. Chen, L. Luo, S. B. Yin. *ACS Sustain. Chem. Eng.,* 2021, **9**, 8249.

[S19] N. U. A. Babar, A. Khan, A. S. Hakeem, H. D. Mohamed, M. H. A. Al-Saeed, M. A. Ehsan. *J. Environ. Chem. Eng*., 2022, **10**, 107959.

[S20] S. F. Zai, A. Q. Dong, J. Li, Z. Wen, C. C. Yang, Q. Jiang. *J. Mater. Chem. A.*, 2021, **9**, 6223.

[S21] F. L. Li, Q. Shao, X. Q. Huang, J. P. Lang. *Angew. Chem. Int. Ed.*, 2018, **57**, 1888.

[S22] K. Dastafkan, S. H. Wang, C. L. Rong, Q. Meyer, Y. B. Li, Q. Zhang, C. Zhao. *Adv. Funct. Mater.*, 2022, **32**, 2107342.

[S23] B. C. Moon, W. H. Choi, K. H. Kim, D. G. Park, J. W. Choi, J. K. Kang, *Small,* 2019, **15**, 1804764.

[S24] Z. H. Wan, Z. Z. Ma, H. F. Yuan, K. Liu, X. G. Wang. *ACS Appl. Energy Mater*., 2022, **5**, 4603.

[S25] C. Tang, N. Y. Cheng, Z. H. Pu, W. Xing, X. P. Sun. *Angew. Chem. Int. Ed.,* 2015, **54**, 9351.

[S26] H. S. Hu, Y. Li, Y. R. Shao, K. X. Li, G. Deng, C. B. Wang, Y. Y. Feng. *J. Power Sources,* 2021, **484**, 229269.

[S27] P. Li, B. Z. Wu, K. F. Du, Z. Liu, E. L. Gao, H. Y. Yin, D. H. Wang. *ACS Sustain. Chem. Eng.,* 2023, **11**, 14246.

[S28] X. Zou, Y. P. Liu, G. D. Li, Y. Y. Wu, D. P. Liu, W. Li, H. W. Li, D. J. Wang, Y. Zhang, X. X. Zou. *Adv. Mater*., 2017, **29**, 1700404.

[S29] Y. Huang, Z. Wang, H. Xiao, Q. Liu, X. Wang. *J. Am. Chem. Soc.,* 2024, **146**, 29006.

[S30] G. Yang, D, Fang, Y. Fu, D. Gao, C. Cheng, J. Li. J. *Colloid Interface Sci.,* 2025, **678**, 717.

[S31] M. Han, C. Wang, J. Zhong, J. Han, N. Wang, A. Seifitokaldani, Y. Yu, Y. Liu, X. Sun, A. Vomiero, H. Liang. *Appl. Catal. B-Environ.,* 2022, **301**, 120764.

[S32] R. Chen, Y. Yang, W. Wu, S. Wu, S. Chen, Z. Wang, Y. Zhu, N. Cheng. *Chem. Eng. J.,* 2024, **480**, 148100.

[S33] J. Wu, Y. Yuan, L. Wang, H. Wang, J. Zheng, B. Jia. *Chem. Eng. J.,* 2025, 164529.
